# Supplementary material for: A comprehensive molecular phylogeny of Geometridae (Lepidoptera) with a focus on enigmatic small subfamilies
Source: PeerJ. 2019 Aug 27;7:e7386. doi: 10.7717/peerj.7386 (PMC6716565; doi:10.7717/peerj.7386)

Appendix S2 of

A comprehensive molecular phylogeny of Geometridae (Lepidoptera) with a focus on enigmatic small subfamilies

Leidys Murillo-Ramos, Gunnar Brehm, Pasi Sihvonen, Axel Hausmann, Sille Holm, Hamid Ghanavi, Erki Õunap, Andro Truuverk, Hermann Staude, Egbert Friedrich, Toomas Tammaru, Niklas Wahlberg

Compiled by G. Brehm

Photos and material by Australian National Insect Collection (ANIC), Egbert Friedrich (EF), Axel Hausmann (AF), B. Christian Schmidt (CS), Pasi Sihvonen (PS), Dirk Stadie (DS); all other photos (including "from XX") by Gunnar Brehm

1 ARCH Archiearinae
2 ARCH > ENNO Archiearinae transferred to Ennominae
3 OENO > STER Oenochrominae transferred to Sterrhinae (see also Sihvonen et al. in preparation)
4 DESM Desmobathrinae
5 EUME *Eumelea*
6 OENO Oenochrominae
7 EPID New subfamily Epidesmiinae

How to read the pages

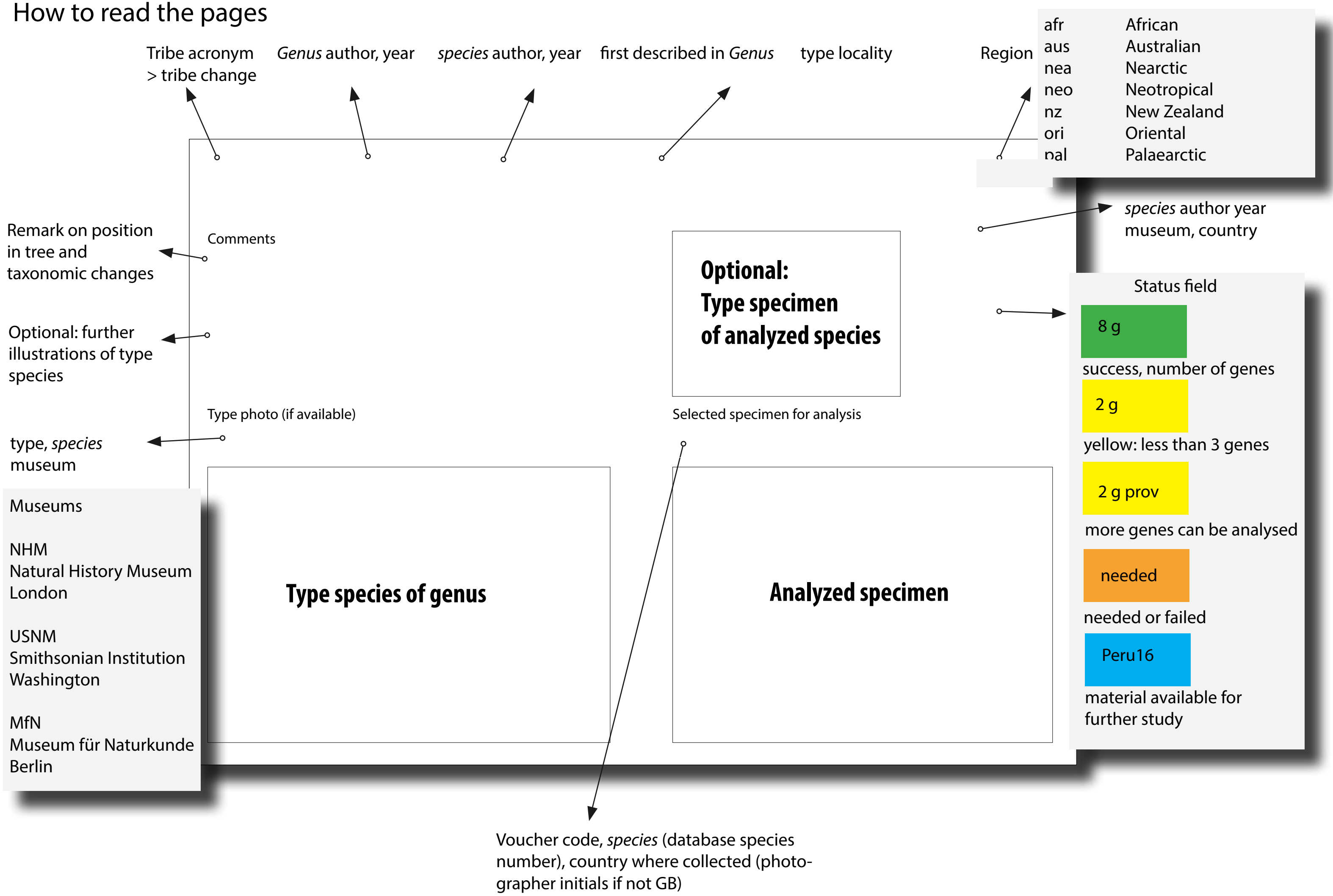

1 Archiearinae

ARCH *Archiearis* Hübner, [1823] *parthenias* Linné, 1761 4 LSL Sweden

nea-pal

Comments

9 g

Type photo (if available)

no type *infans* NHM

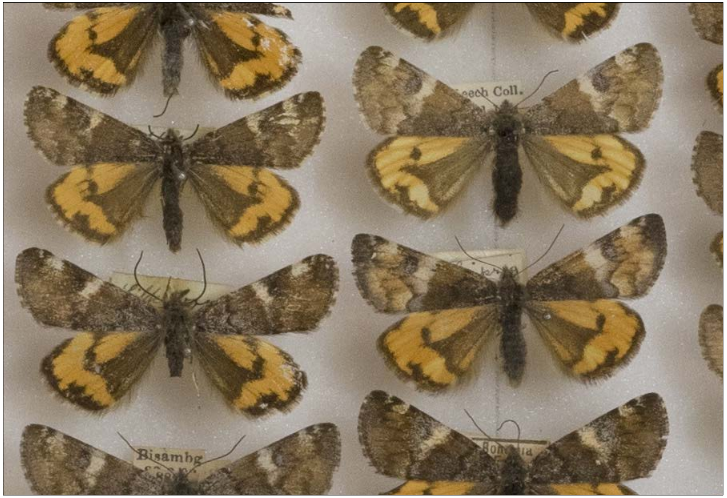

Selected specimen for analysis

CNC508477 *A. infans* Canada (from CS)

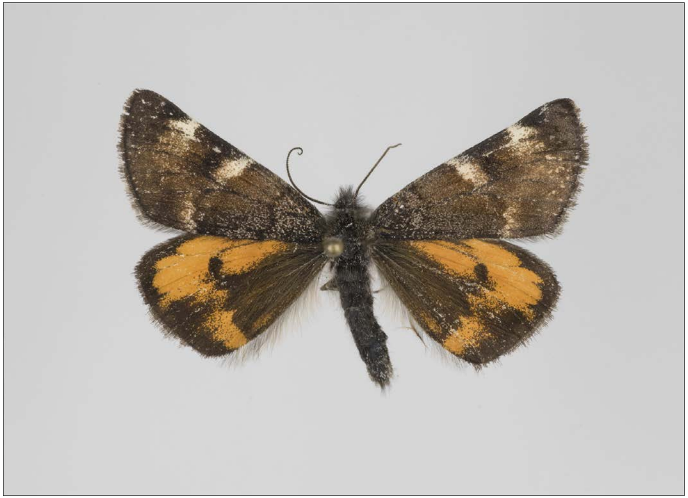

ARCH *Archiearis* Hübner, [1823] *parthenias* Linné, 1761 4 LSL Sweden

nea-pal

Comments

9 g

Type photo (if available)

no type *parthenias* NHM

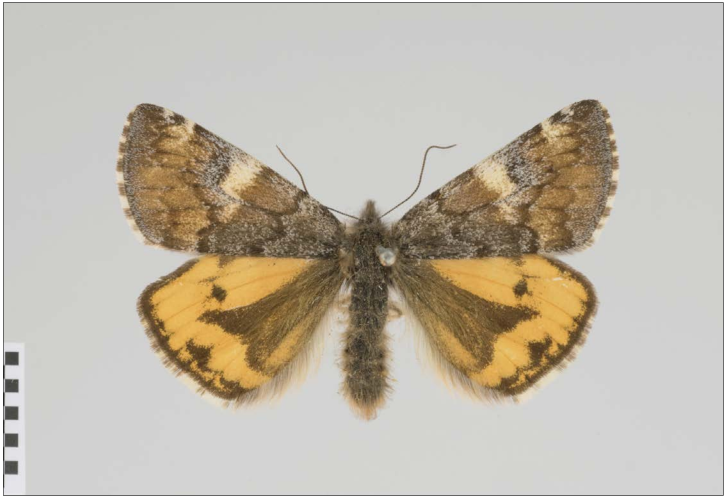

Selected specimen for analysis

NW107-1 *A. parthenias* Sweden (NW)

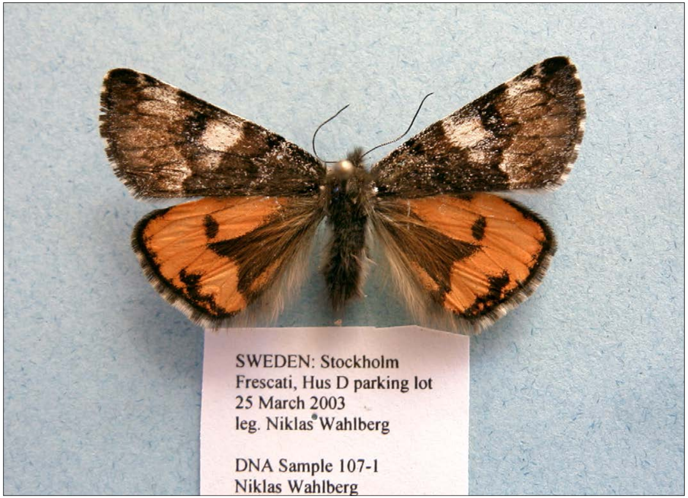

ARCH *Boudinotiana* Leraut, 2002 check *notha?* unclear deposit pal

Comments

5 g

Type photo (if available)

no type *notha* BC_ZSM_Lep_82203

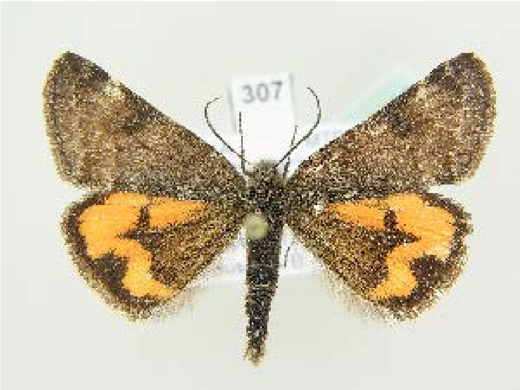

Selected specimen for analysis

EO0196 no photo

ARCH *Leucobrephos* Grote, 1874 *brephoides* Walker, 1857 3 NHM Canada: Hudson Bay

nea-pal

Comments

6 g

Type photo (if available)

type *brephoides* NHM

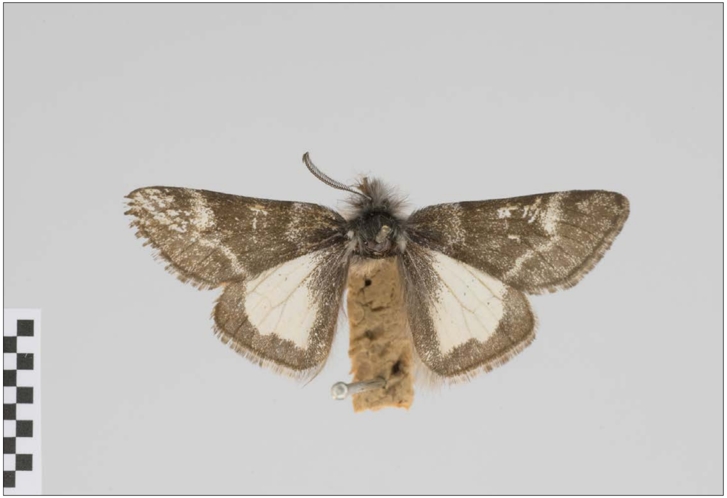

Selected specimen for analysis

CNC508469 *L. brephoides* Canada (from CS)

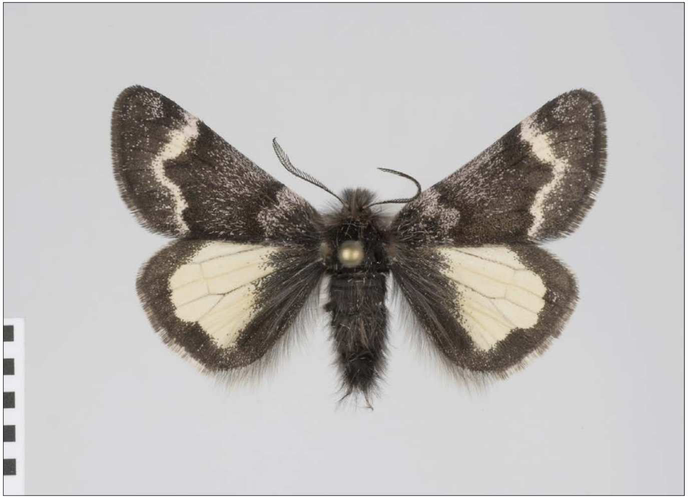

2 Archiearinae transferred to Ennominae

ARCH *Acalyphes* Turner, 1926 *philorites* Turner, 1926 1 unclear deposit Tasmania aus

Comments

Young (2006): not Archiearinae
transferred to Ennominae

2 g

Type photo (if available)

no type *A. philorites* 10ANIC_12135

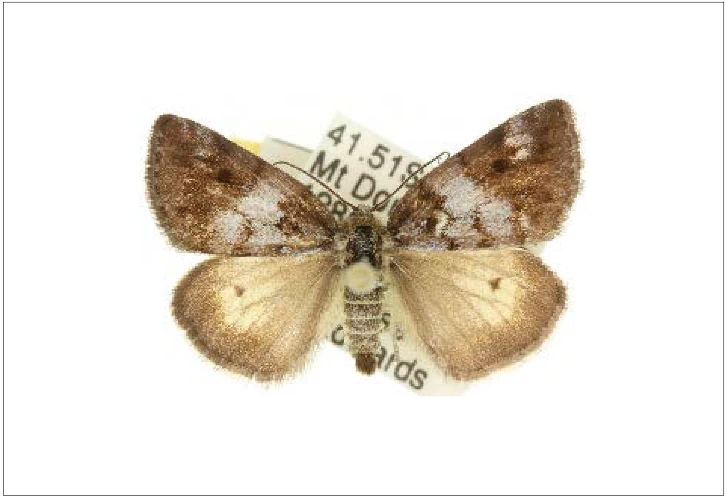

Selected specimen for analysis

10ANIC-12134 *A. philorites* Australia

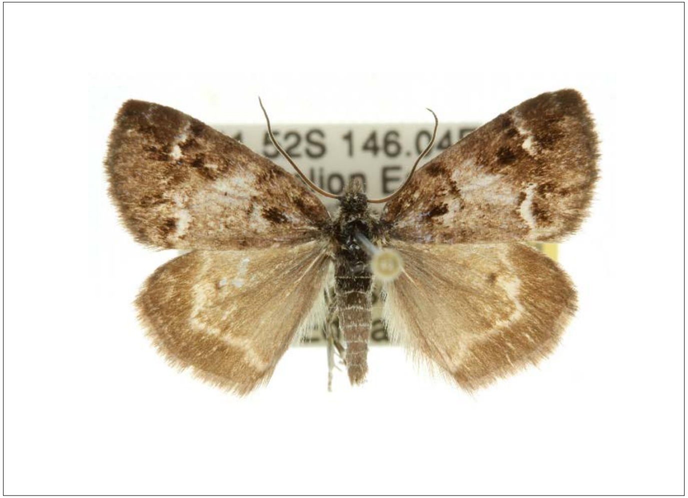

ARCH *Dirce* Prout, 1910 *lunaris* Meyrick, 1890 4 NHM Tasmania

aus

Comments

Young (2006): not Archiearinae
transferred to Ennominae

2 g

Type photo (if available)

Seitz illustration *lunaris*

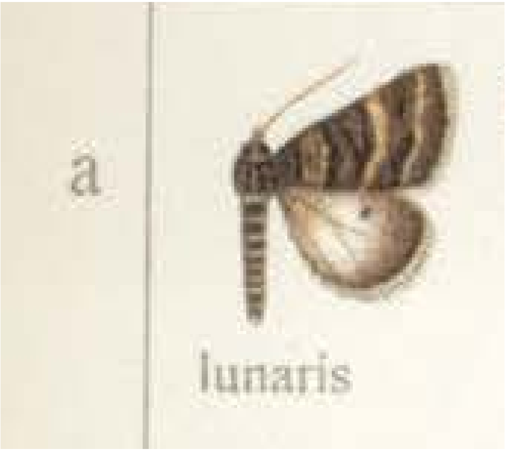

Selected specimen for analysis

10ANIC-12152 *D. aesiodora* Australia

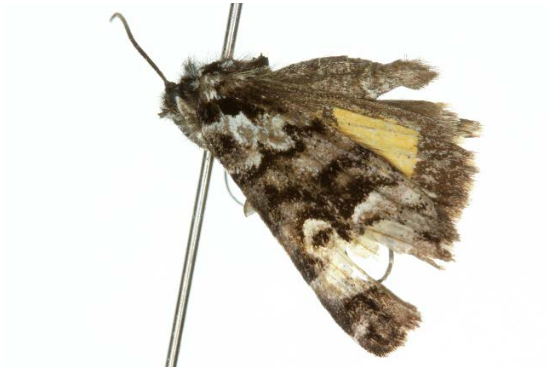

3 Oenochrominae transferred to Sterrhinae

OENO > STER *Ametris* Hübner, [1822] 1806 *nitocritaria* Hübner, 1822 2 unclear deposit ? neo

Comments

transferred to Sterrhinae
(Sihvonen et al. in prep.)

9 g

Type photo (if available)

Hübner illustration *nitocris*

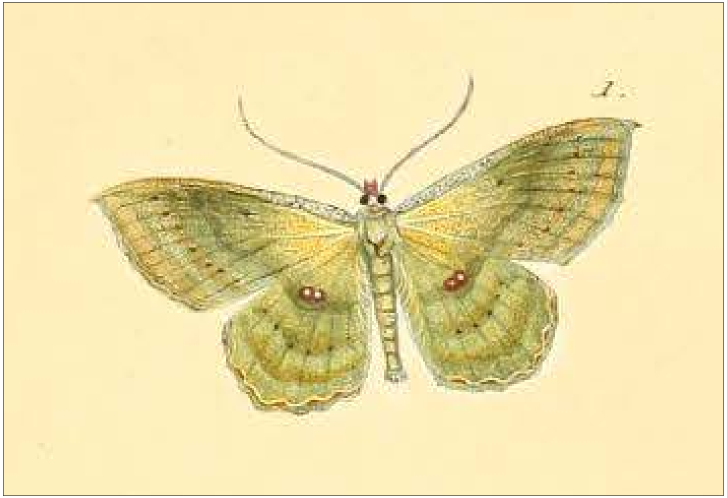

Selected specimen for analysis

CNCLEP00152613 *A. nitocris* USA (from CS)

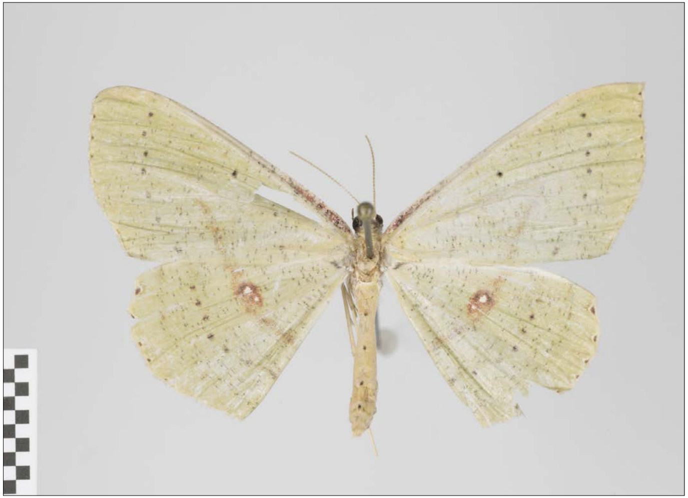

OENO > STER *Ergavia* Walker, 1866 *bogotaria* Walker, 1866 22 NHM Colombia Bogota

neo

Comments

transferred to Sterrhinae
(Sihvonen et al. in prep.)

7 g

Type photo (if available)

type *bogotaria* NHM (syn of *carinenta* Cramer)

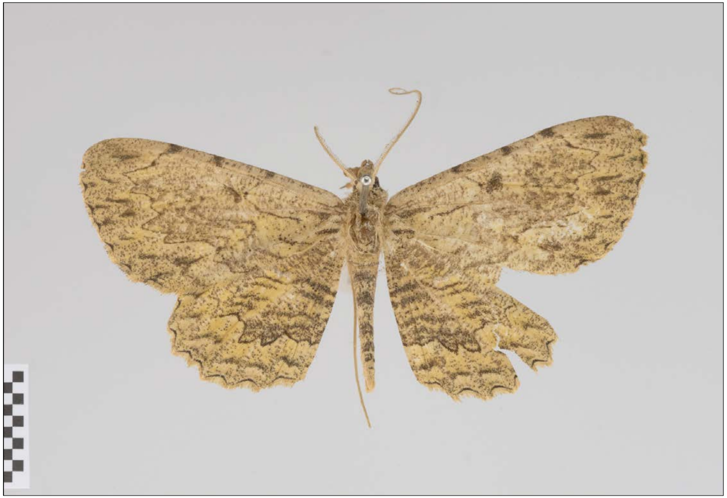

Selected specimen for analysis

ID 17186 *Ergavia* sp Ecuador

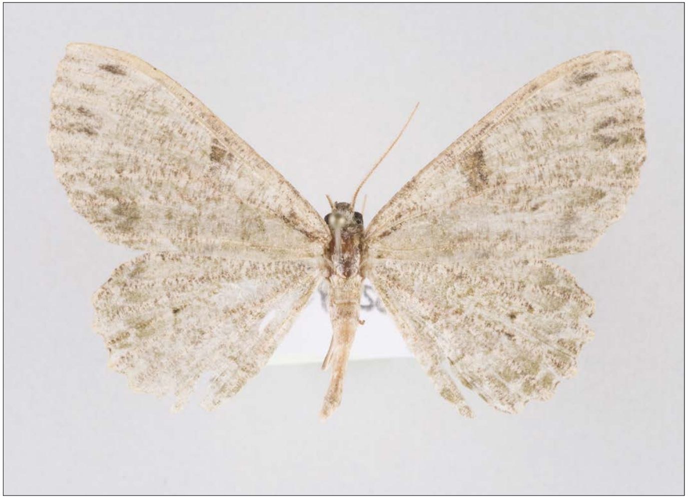

OENO > STER *Macrotes* Westwood, 1841 *netrix* Cramer, 1777 3 unclear deposit Guyana or near neo

Comments

transferred to Sterrhinae
(Sihvonen et al. in prep.)

7 g

Type photo (if available)

Cramer illustration *netrix*

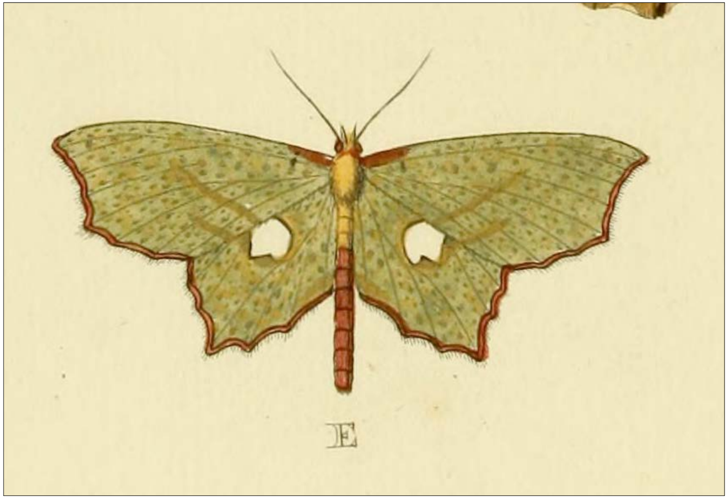

Selected specimen for analysis

ID 22654 *M. netrix* (#2669) Ecuador

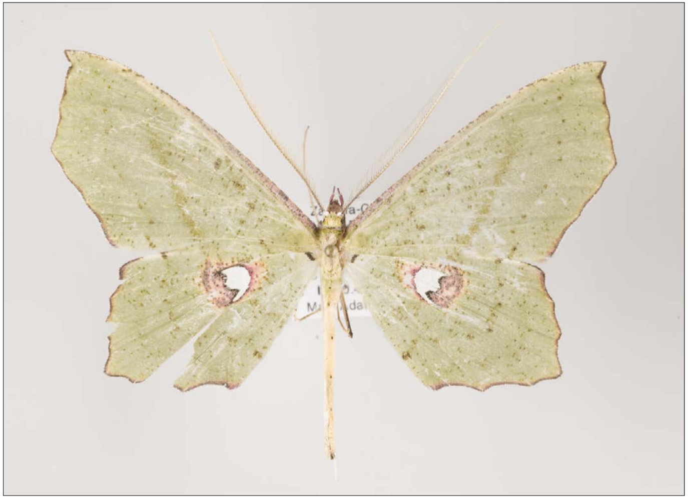

5 Desmobathrinae

DESM *Conolophia* Warren, 1894 *conscitaria* Walker, 1861 8 NHM Congo afr-mad-ori

Seitz illustration

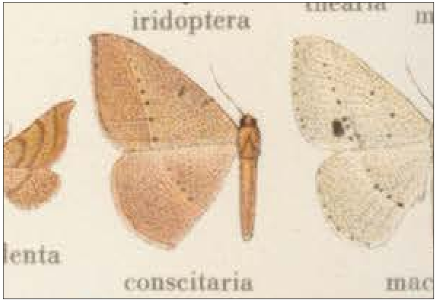

6 g

Type photo (if available)

type *conscitaria* NHM

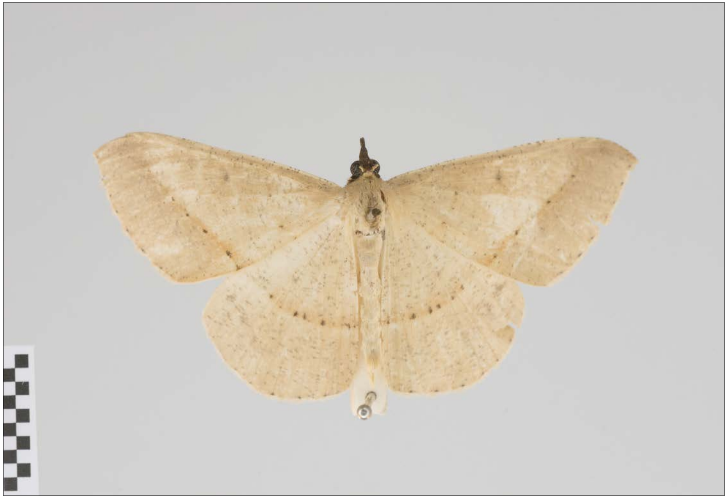

Selected specimen for analysis

MM00224 *C.* nr *conscitaria* South Africa (PS)

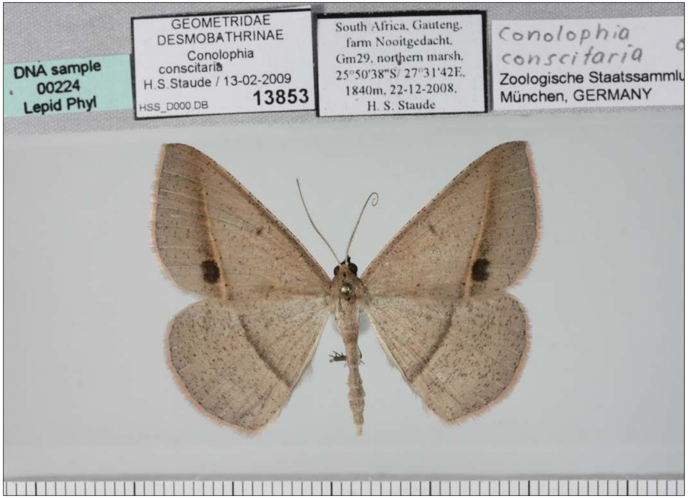

DESM *Conolophia* Warren, 1894 *conscitaria* Walker, 1861 8 NHM Congo afr-mad-ori

Seitz illustration

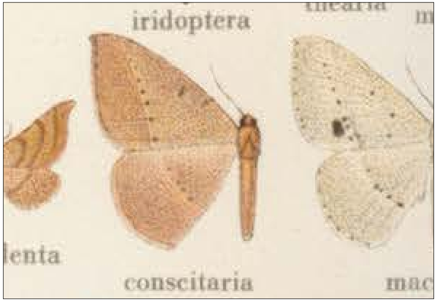

10 g

Type photo (if available)

type *conscitaria* NHM

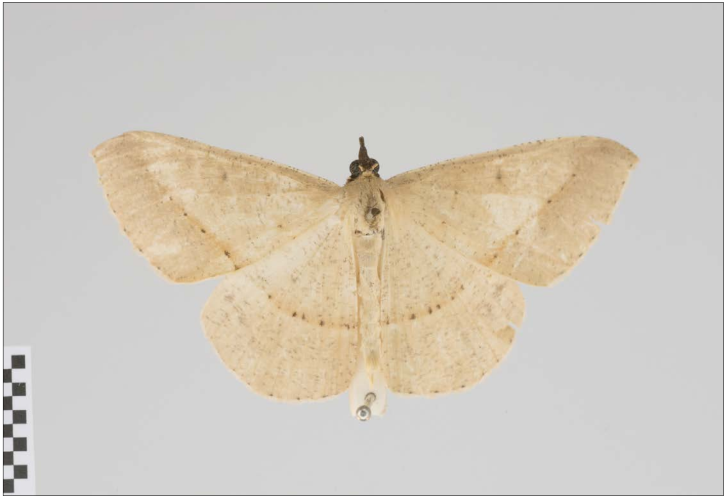

Selected specimen for analysis

PS265 *C. conscitaria* South Africa (PS)

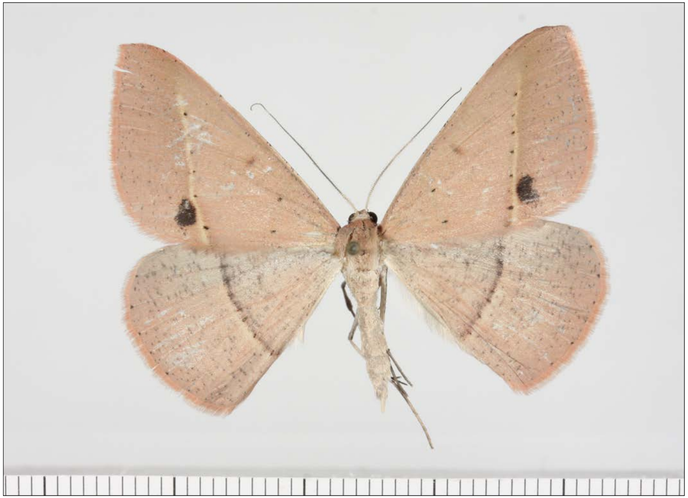

DESM *Derambila* Walker, [1863] *punctisignata* Walker, 1863 44 OUM West Africa afr-mad-aus-oce-pal-nea

Comments

7 g

Type photo (if available)

type *punctisignata* NHM

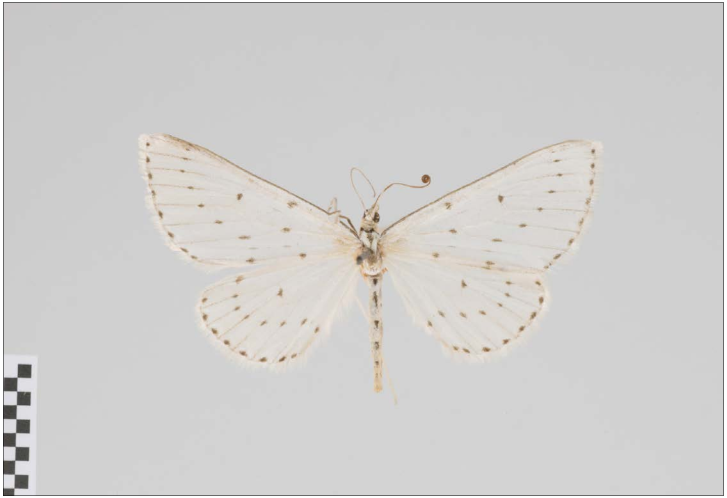

Selected specimen for analysis

PS182 *D.* nr *saponaria* Asia (PS)

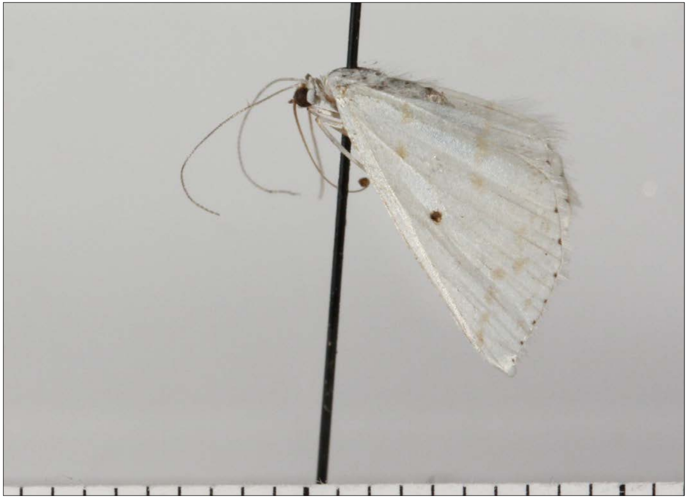

DESM *Dolichoneura* Warren, 1894 *albidentata* Warren, 1894 11 NHM Guyana neo

Comments

Seitz illustration

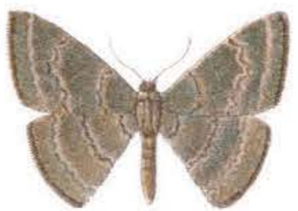

7 g

Type photo (if available)

no type *albidentata* NHM

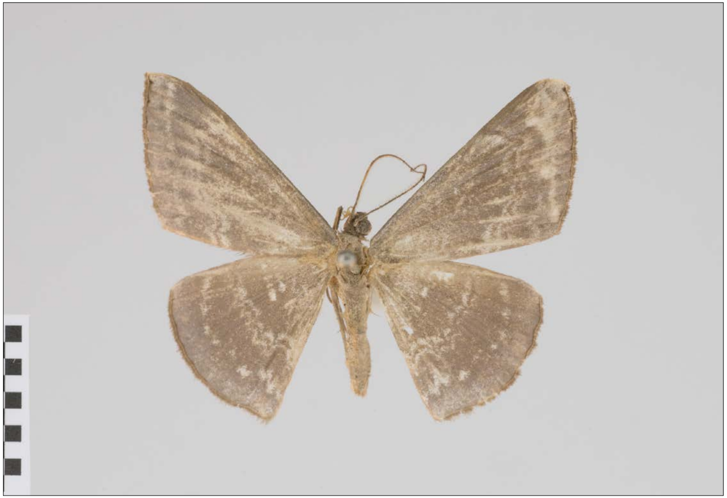

Selected specimen for analysis

AH7160 *D. oxypteraria* Peru (from AH)

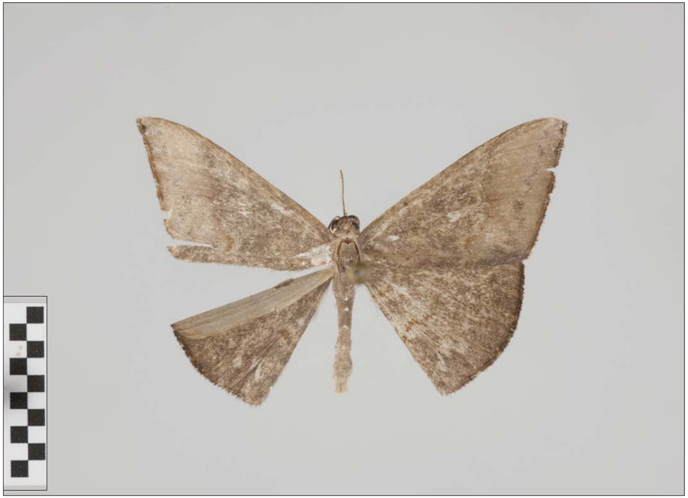

DESM *Noreia* Walker, 1861 *perdensata* Walker, 1862 13 NHM Sri Lanka

aus-ori

Comments

6 g

Type photo (if available)

no type *perdensata* NHM

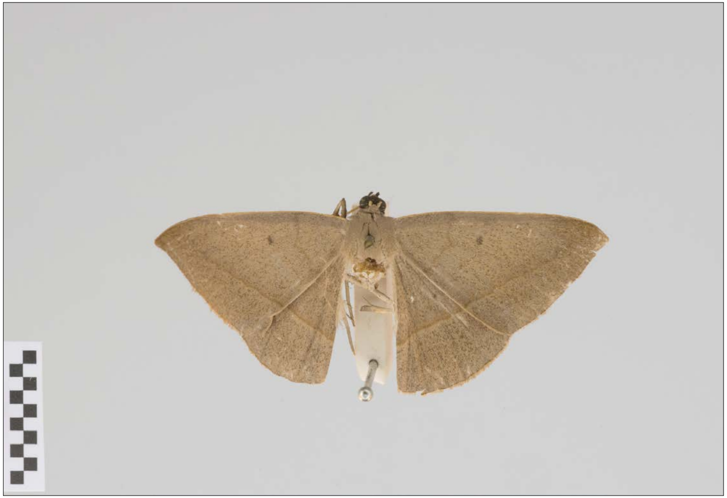

Selected specimen for analysis

PS183 *Noreia* sp Asia (PS)

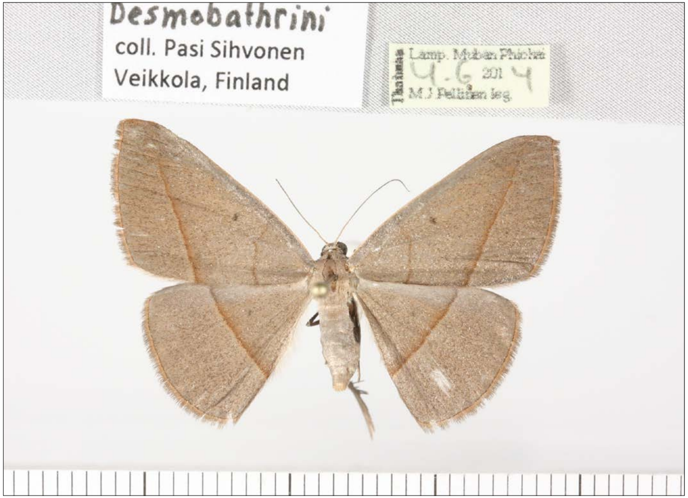

DESM *Ophiogramma* Hübner, [1831] *injunctaria* Hübner, [1831] 8 unclear deposit Br: Rio de Janeiro neo

illustration Hübner

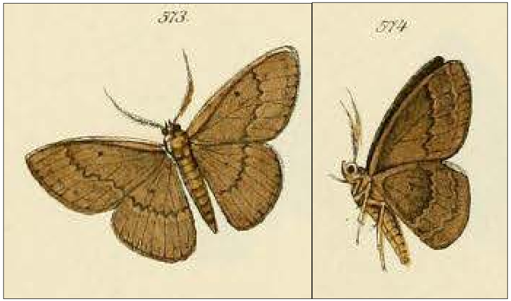

8 g

Type photo (if available)

no type *injunctaria* NHM

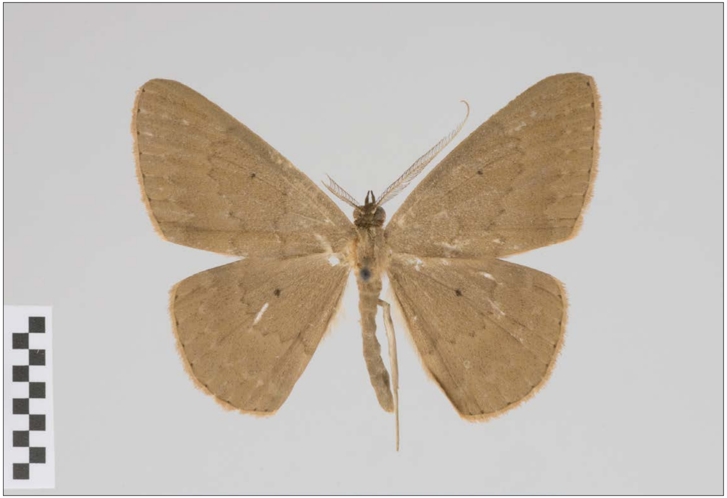

Selected specimen for analysis

gb_ID_19219 *O. coenobiata* (#996) Ecuador

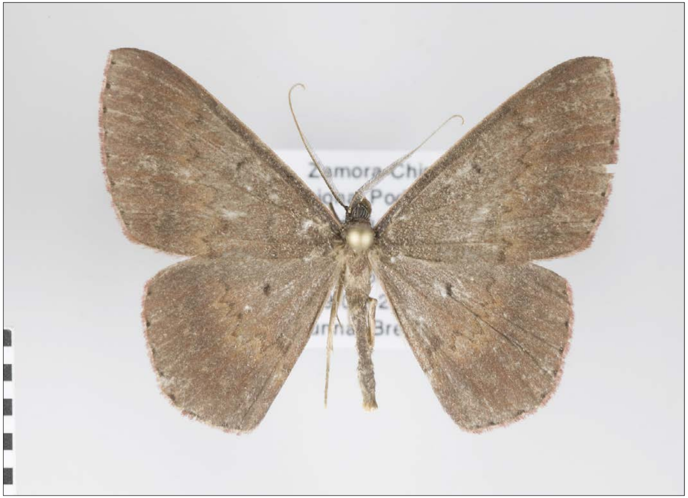

DESM *Ozola* Walker,1861 *microniaria* Walker, 1862 48 NHM Sri Lanka afr-aus-oce-ori-pal

Comments

4 g

Type photo (if available)

Seitz illustration *microniaria*

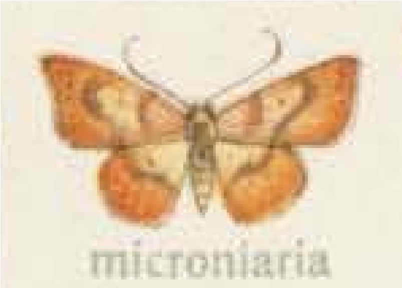

Selected specimen for analysis

PS191 *Ozola* nr *convergens* Thailand (PS)

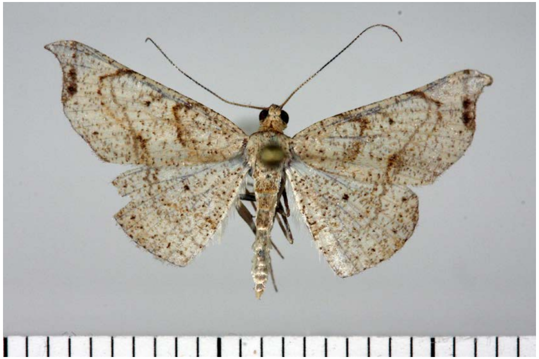

DESM *Ozola* Walker,1861 *microniaria* Walker, 1862 48 NHM Sri Lanka afr-aus-oce-ori-pal

holotype *hollowayi* Scoble & Sommerer, 1988 NHM

Comments

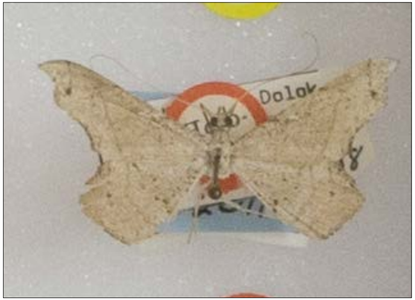

5 g

Type photo (if available)

Selected specimen for analysis

BC86106 *O. hollowayi* Australia (from EF)

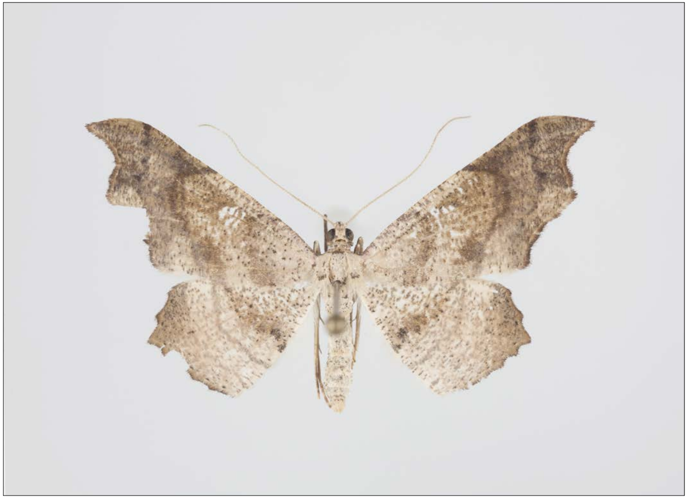

DESM *Ozola* Walker,1861 *microniaria* Walker, 1862 48 NHM Sri Lanka
Seitz illustration *niphoplaca* (type species of Desmobathrinae), described as *Desmobathra* Meyrick, 1886

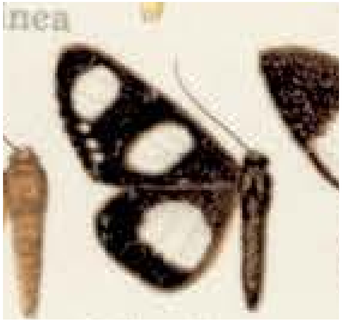

needed

Type photo (if available)

type *niphoplaca* NHM

Selected specimen for analysis

no sequenced material

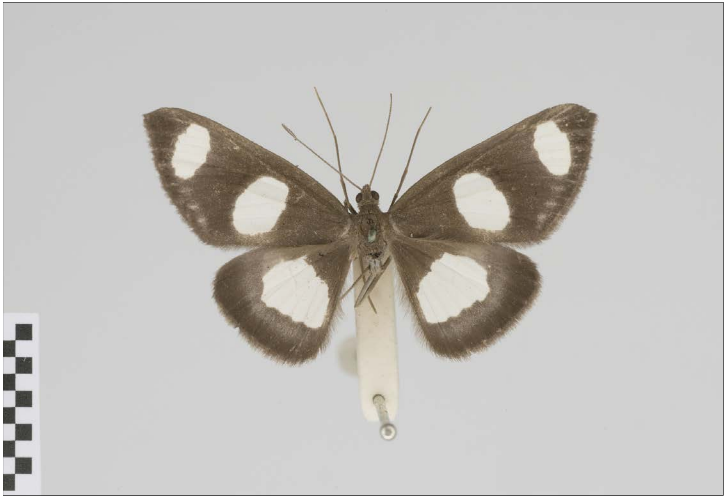

DESM *Pycnoneura* Warren, 1894 *rectilineata* Warren, 1906 9 USNM French Guiana

neo

Seitz illustration

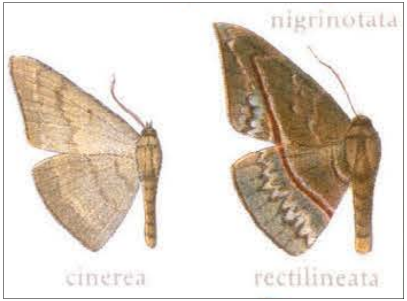

8 g

Type photo (if available)

type *rectilineata* USNM

Selected specimen for analysis

Pe-Geo-1327 *P. rectilineata* Peru

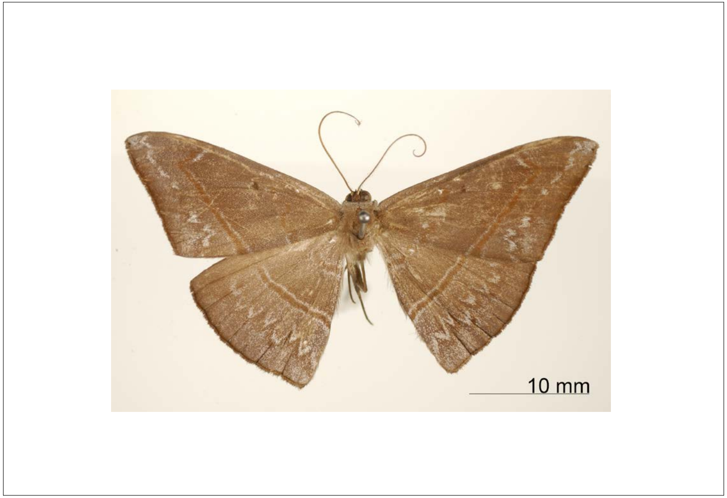

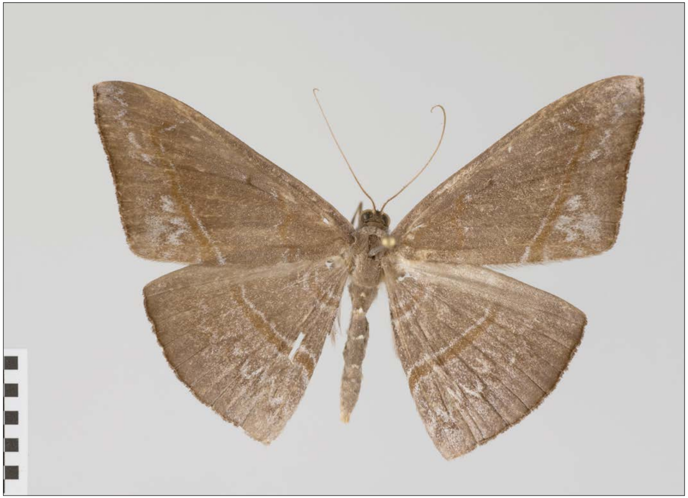

OENO > DESM *Racasta* Walker, 1861 *caberaria* Walker, 1861 2 NHM Venezuela

neo

Comments

transferred to Desmobathrinae

8 g

Type photo (if available)

type *spatiaria caberaria* NHM

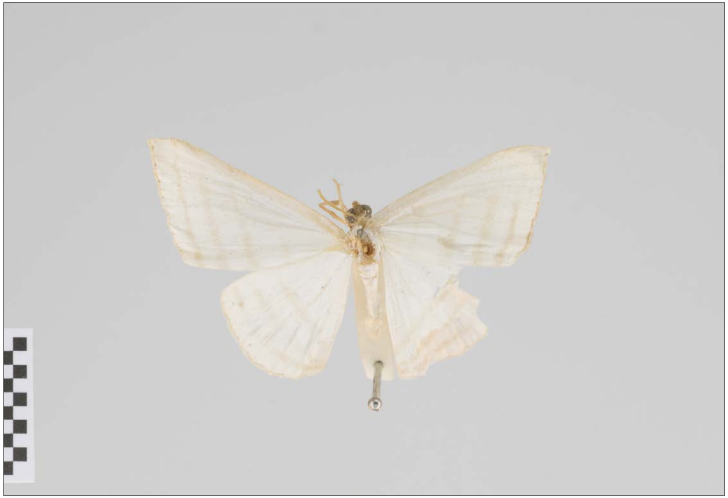

Selected specimen for analysis

gb_ID_19265 *R. spatiaria extendata* (#2676) Ecuador

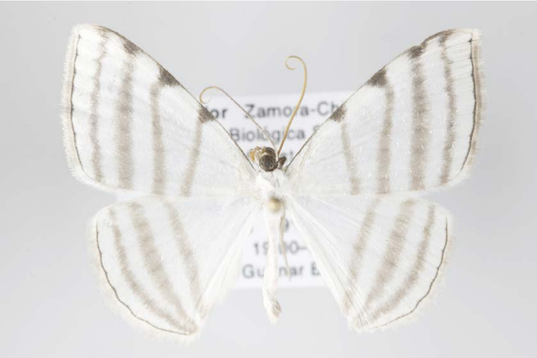

OENO *Nearcha* Guest, 1887 *buffalaria* Guenée, 1887 13 MNHM Tasmania aus

Comments

no photo but likely that this is *N. dasyzona* (barcode checked on BOLD)

transferred to Desmobathrinae?

5 g

Type photo (if available)

type *buffalaria* NHM

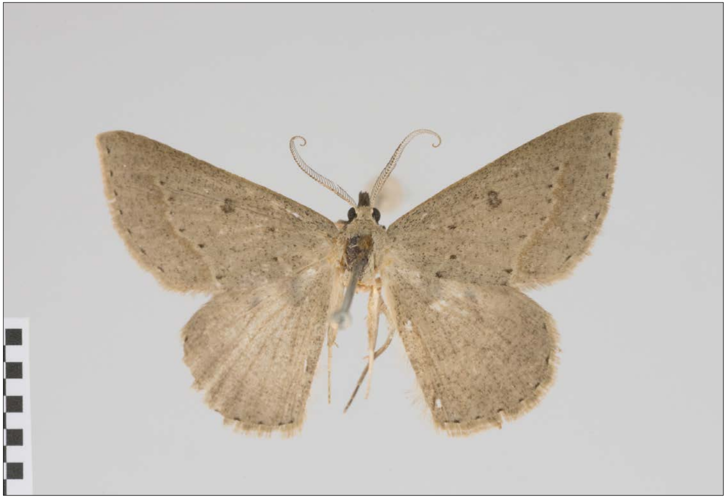

Selected specimen for analysis

MJM_96_0267 *N. dasyzona* no photo

OENO > DESM *Zanclopteryx* Herrich-Schäffer, [1855] *aculeataria* HS, [1855] 8 unclear deposit neo

Comments

transferred to Desmobathrinae

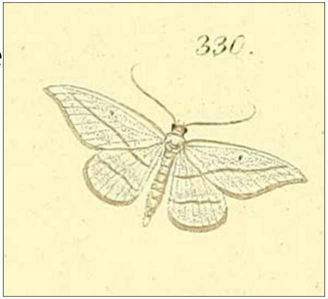

Herrich-Schäffer illustration

8 g

Type photo (if available)

no type *aculeataria* NHM

Selected specimen for analysis

Pe-Geo-0492 *Zanclopteryx* sp Peru

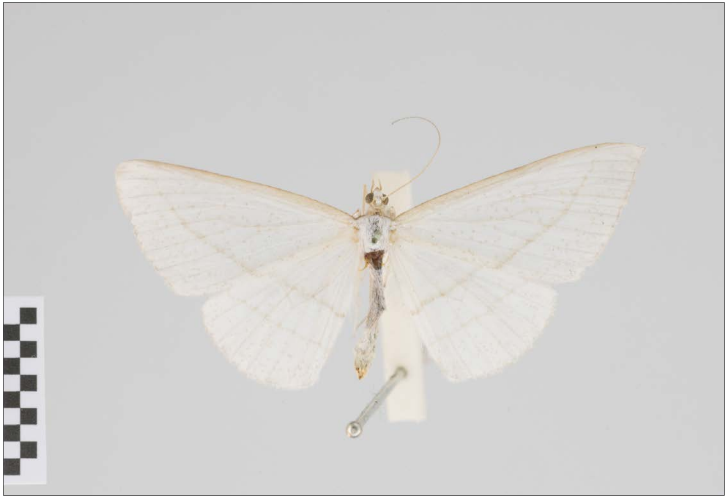

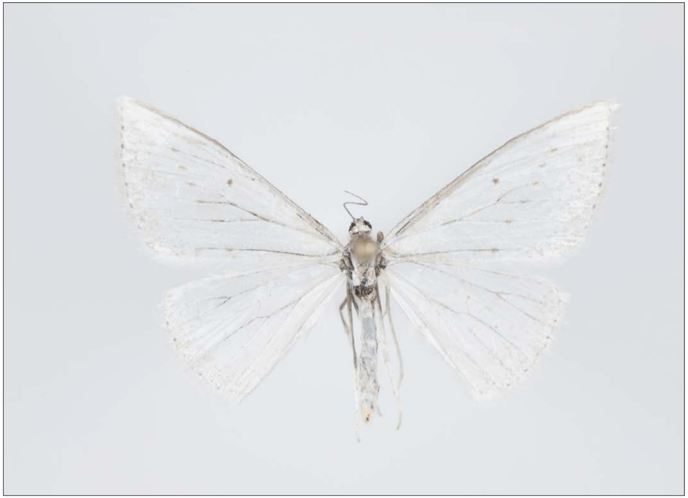

5 *Eumelea*

DESM > UN *Eumelea* Duncan [& Westwood], 1841 *rosalia* Stoll, 1781 28 unclear deposit aus-oce-ori-pal

Indonesia: Moluccas, Amboinia

Comments

transferred to unassigned

Seitz illustration

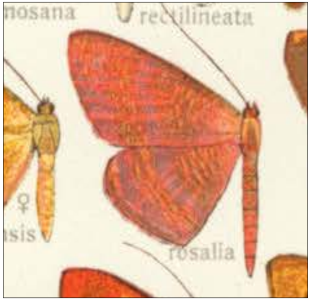

type *ludovicata* Guenée, [1858] NHM Ceylon

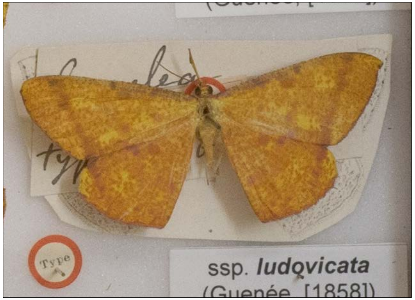

6 g

Type photo (if available)

type *rosalia* NHM

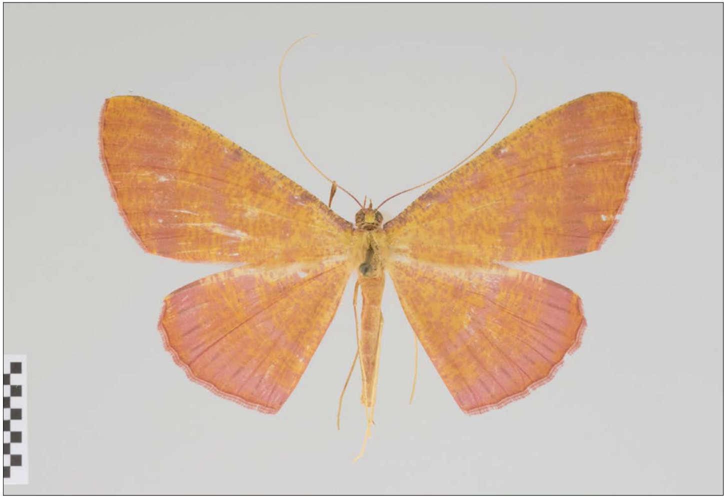

Selected specimen for analysis

MM13433 *E. ludovicata*

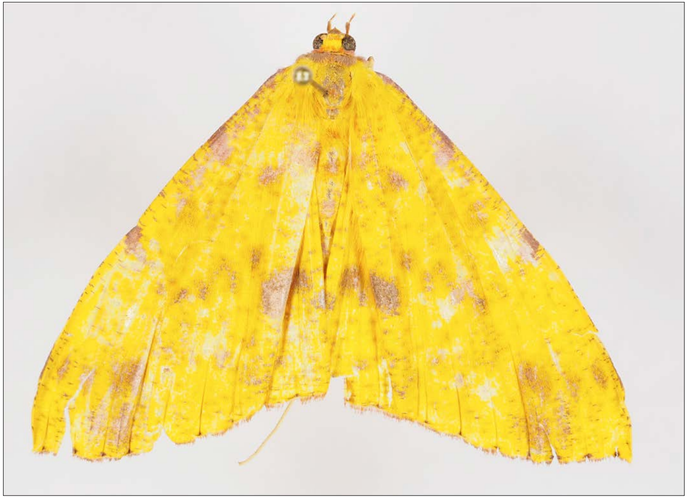

6 Oenochrominae

OENO *Antictenia* Prout, 1910 *punctunculus* Lucas, 1892 2 unclear deposit Austrlia QLD aus

Comments

10 g

Type photo (if available)

Seitz illustration *punctunculus*

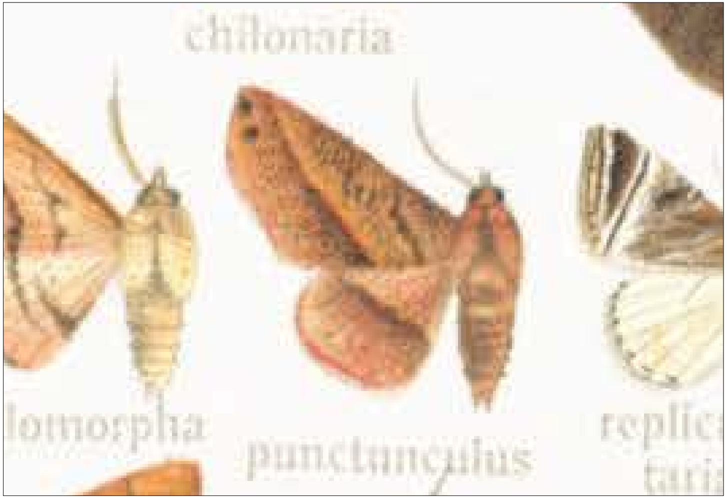

Selected specimen for analysis

EF_Aus-Geo_5 *A. torta* Australia (EF)

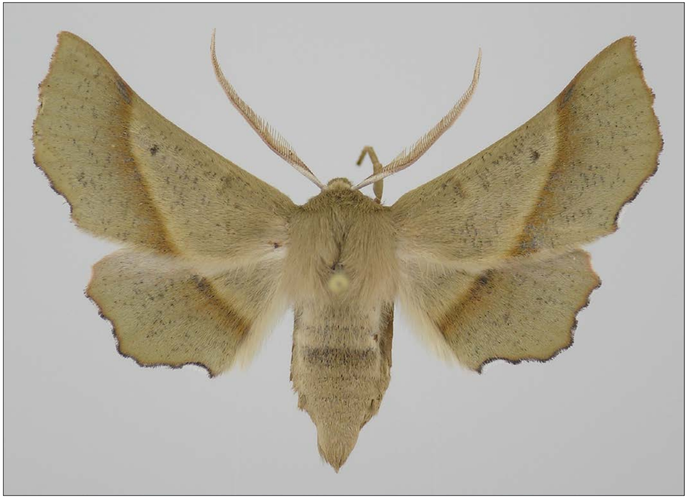

OENO *Arhodia* Guenée, [1858] *lasiocamparia* Gn, 1858 1 MNHN Tasmania

aus

Comments

8 g

Type photo (if available)

type *lasiocamparia* NHM

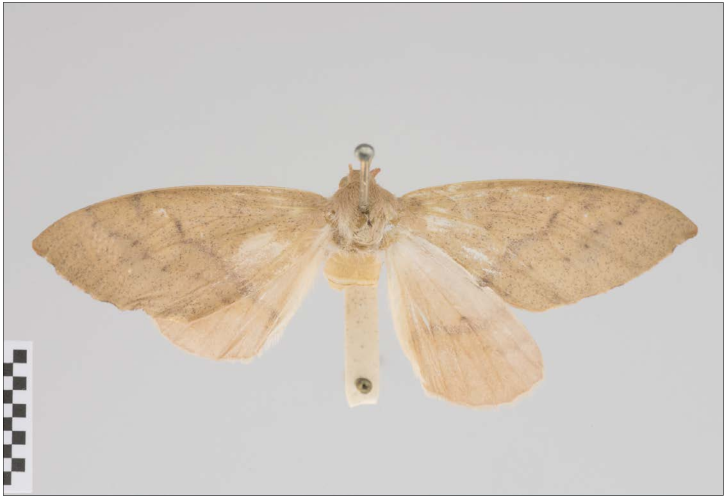

Selected specimen for analysis

EF_Aus-Geo_6 *A. lasiocamparia* Australia

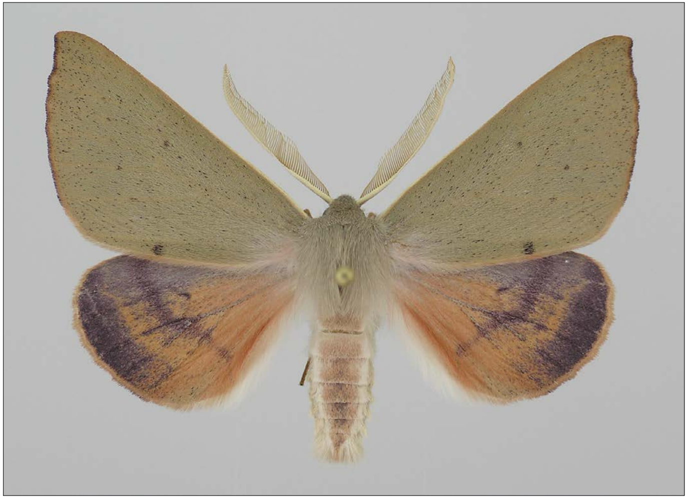

OENO *Dinophalus* Prout, 1910 *cyanorrhoea* Lower, 1903 15 unclear deposit Australia: No. Territory aus

Seitz illustration *serpentaria*

Comments

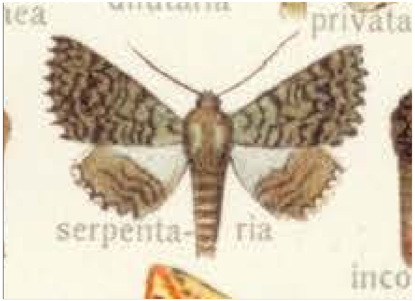

5 g

Type photo (if available)

Selected specimen for analysis

EF_Aus-Geo_9 *D. serpentaria* Australia (EF)

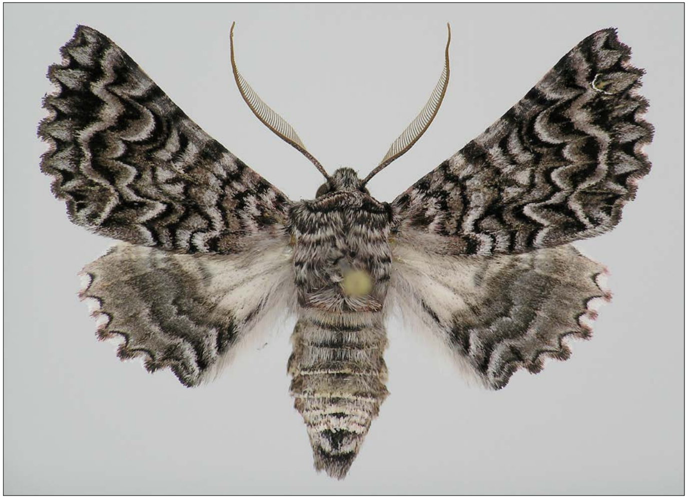

OENO *Gastrophora* Guenée, [1858] *henricaria* Guenée, [1858] 1 NHM Australia

aus

Comments

7 g

Type photo (if available)

type *henricaria* NHM (female)

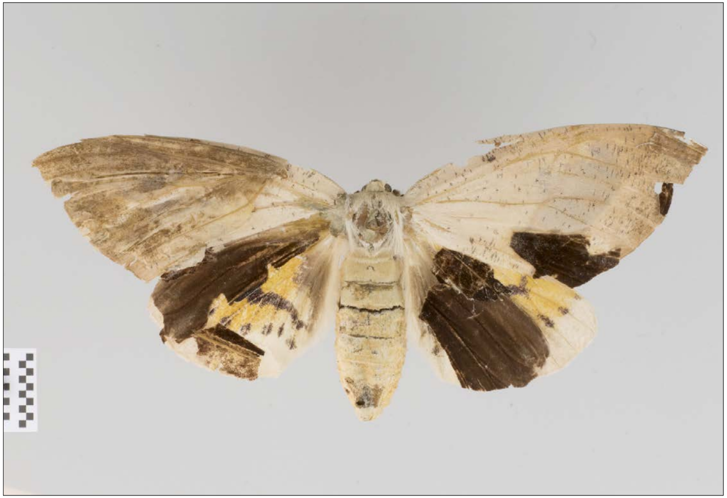

Selected specimen for analysis

EF_Aus-Geo_1 *G. henricaria* Australia (EF)

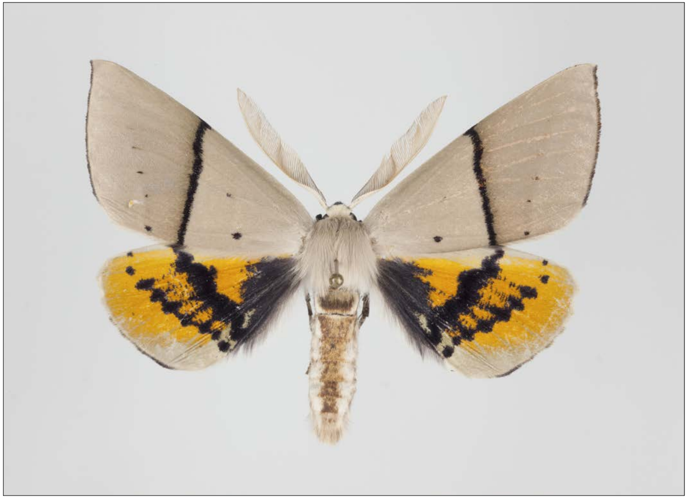

OENO *Homospora* Turner, 1904 *procrita* Turner, 1904 1 unclear deposit Austrlia QLD aus

Seitz illustration *rhodoscopa*

Comments

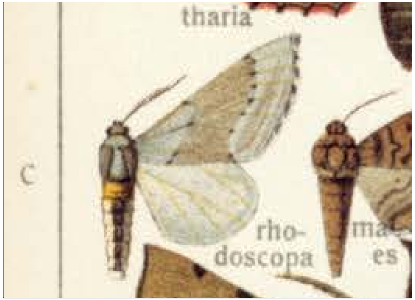

9 g

Type photo (if available)

Selected specimen for analysis

EF_Aus-Geo_4 *H. rhodoscopa* Australia (EF)

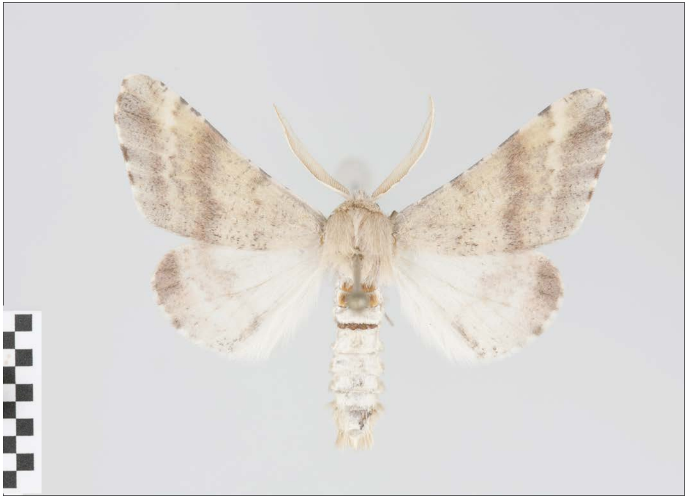

OENO *Hypographa* Guenée, [1858] *phlegetonaria* Guenée, [1858] 4 MNHM Tasmania neo

Comments

9 g

Type photo (if available)

Paralectotype *H. phlegetonaria* NHM

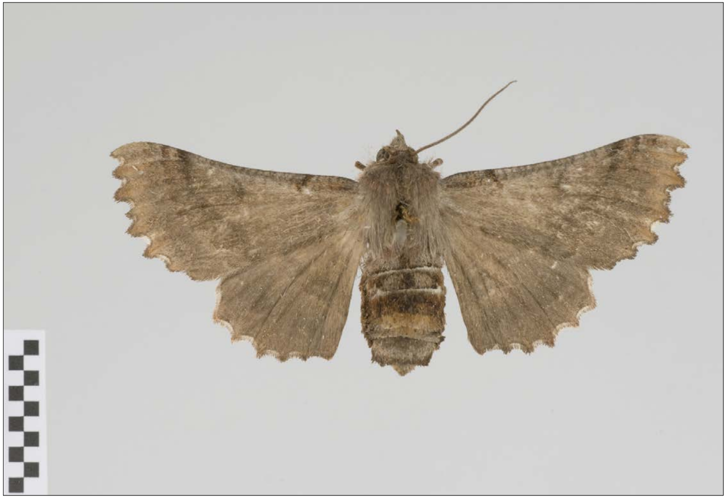

Selected specimen for analysis

EF_Aus-Geo_13 *H. epiodes* Australia (EF)

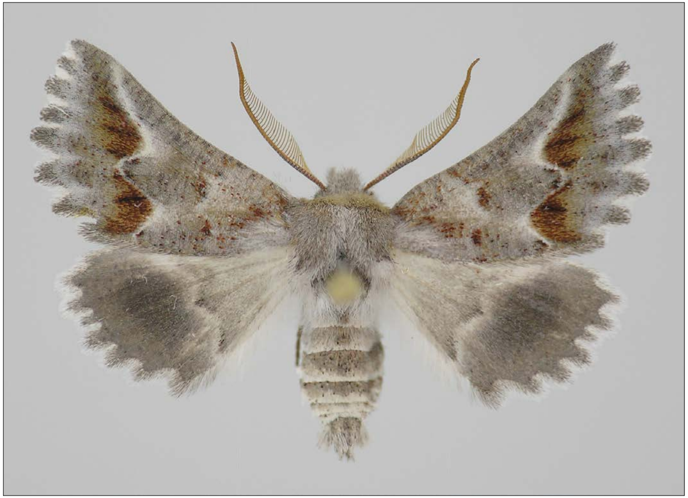

OENO *Lissomma* Warren, 1905 *himerata* Warren, 1905 4 NHM Australia N Territory of S Australia aus

Comments

Lissomma dilutaria in Epidesmiinae

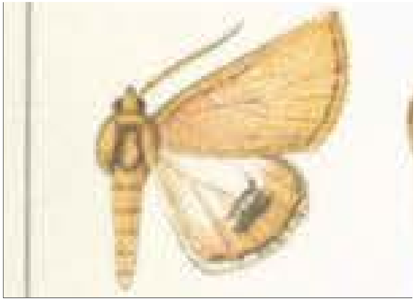

9 g

Type photo (if available)

type *L. himerata* NHM

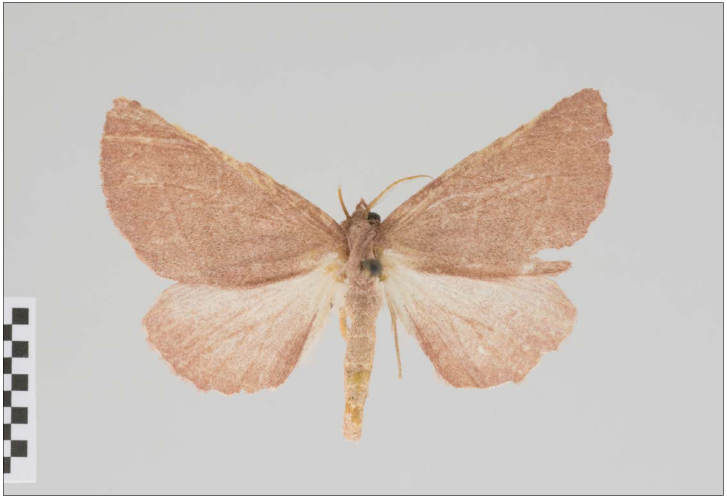

Selected specimen for analysis

EF_Aus-Geo_14 *L. postcarneata* Australia (EF)

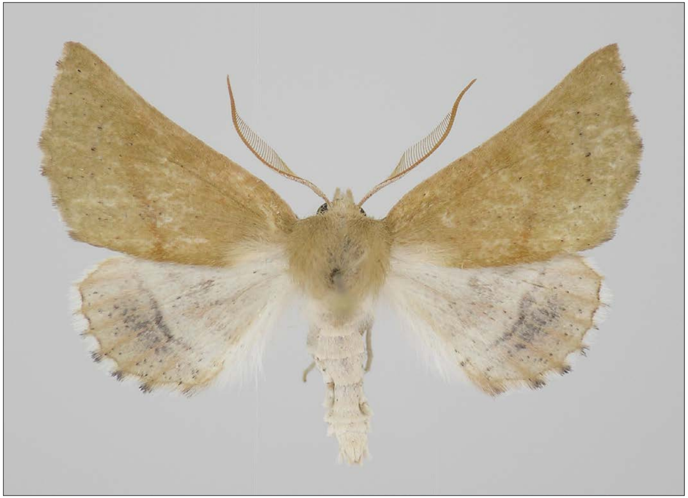

OENO *Monoctenia* Guenée, [1858] *falernaria* Gn, [1858] 2 MNHM Tasmania

aus

Comments

10 g

Type photo (if available)

no type *M. falernaria* NHM

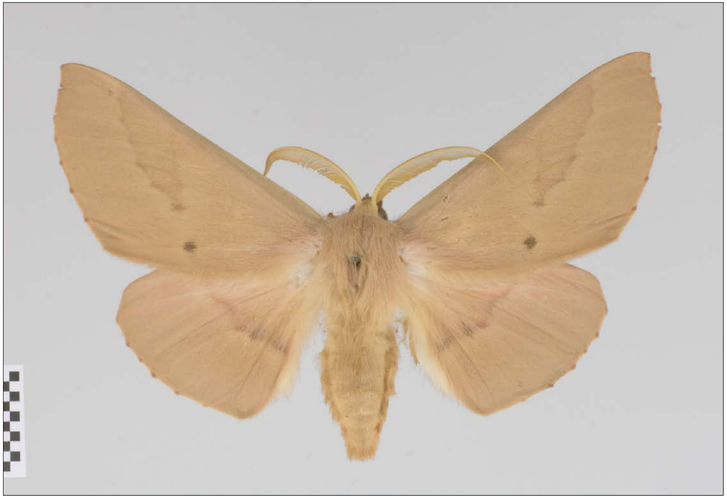

Selected specimen for analysis

EF_Aus-Geo_15 *Monoctenia* sp Australia (EF)

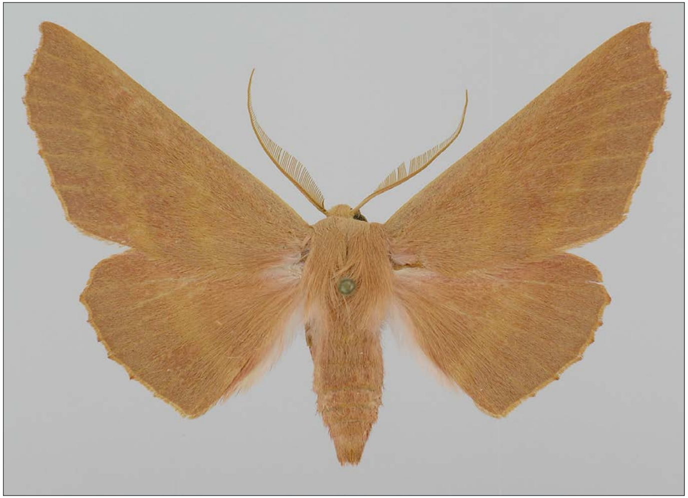

OENO *Oenochroma* Guenée, [1858] *vinaria* Guenée, [1858] 20 MNHM Australia

aus

Comments

forms a clade with *vinaria*

8 g

Type photo (if available)

no type *vinaria* NHM

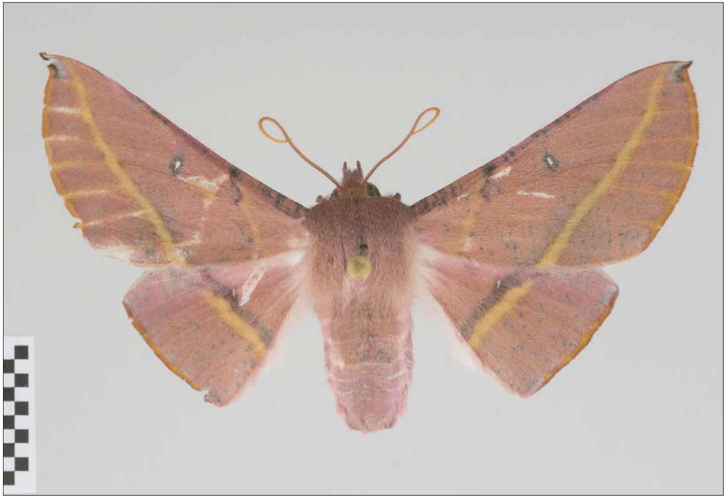

Selected specimen for analysis

MM07576 *O. orthodesma* Australia

photo of specimen misses

OENO *Oenochroma* Guenée, [1858] *vinaria* Guenée, [1858] 20 MNHM Australia

aus-oce

Comments

forms a clade with *orthodesma*

2 g

Type photo (if available)

no type *vinaria* NHM

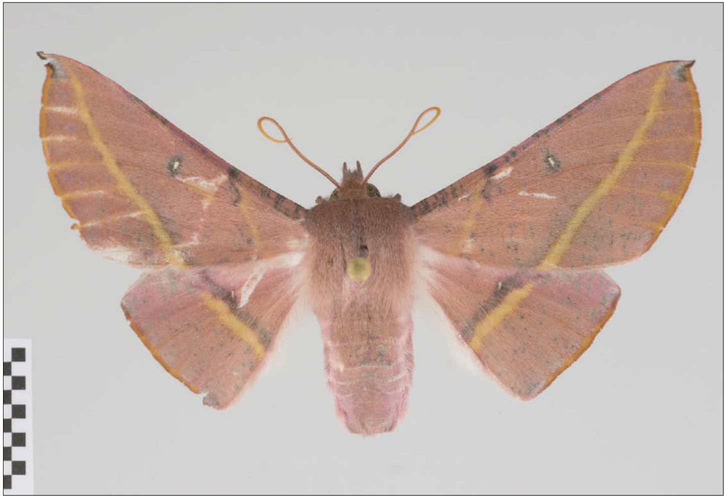

Selected specimen for analysis

10ANIC_11131 *O. vinaria* Australia

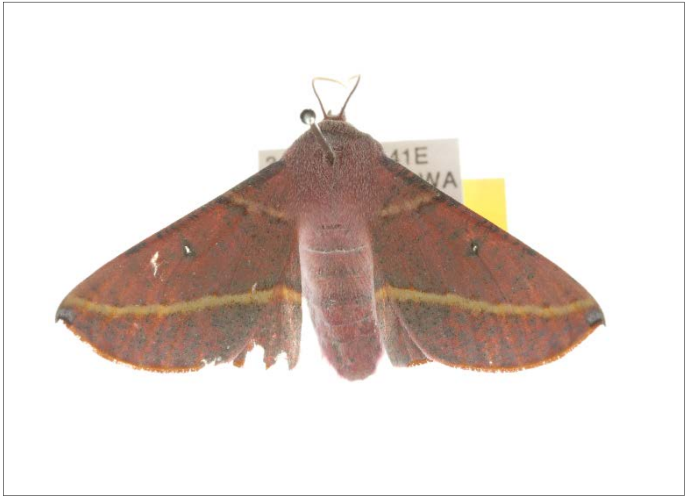

OENO *Oenochroma* Guenée, [1858] *vinaria* Guenée, [1858] 20 MNHM Australia

aus-oce

Comments

needs to be excluded from *Oenochroma*

6 g

Type photo (if available)

Seitz illustration

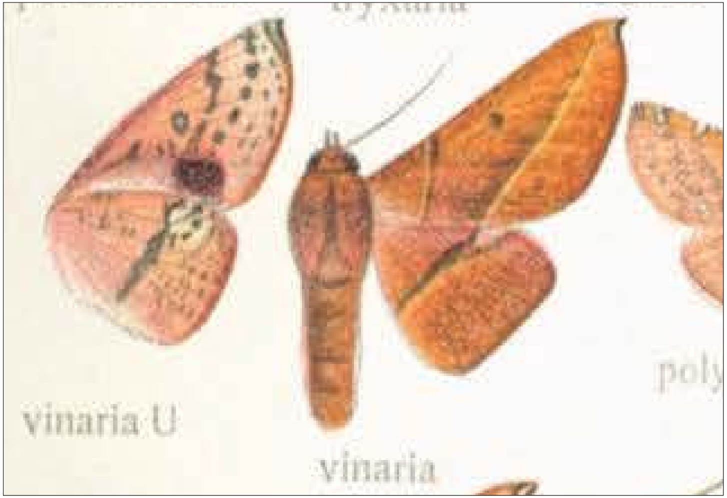

Selected specimen for analysis

EF_Aus-Geo_022 *O. infantilis* Australia (EF)

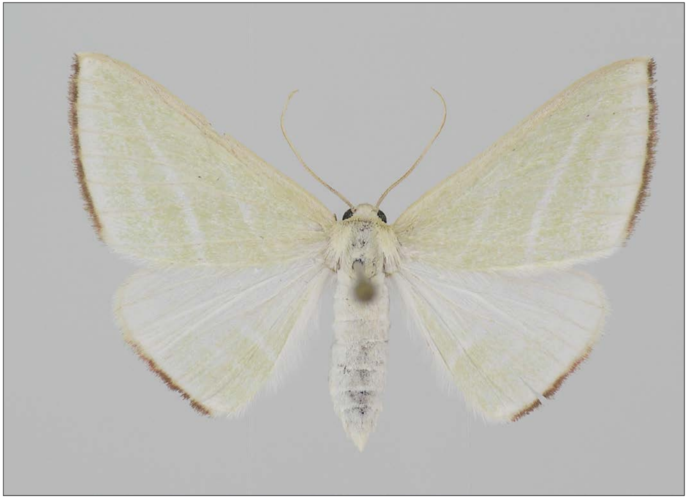

OENO *Onycodes* Guenée, [1858] *traumataria* Guenée, [1858] 3 MNHM Australia

afr-aus

Comments

8 g

Type photo (if available)

Seitz illustration *traumataria*

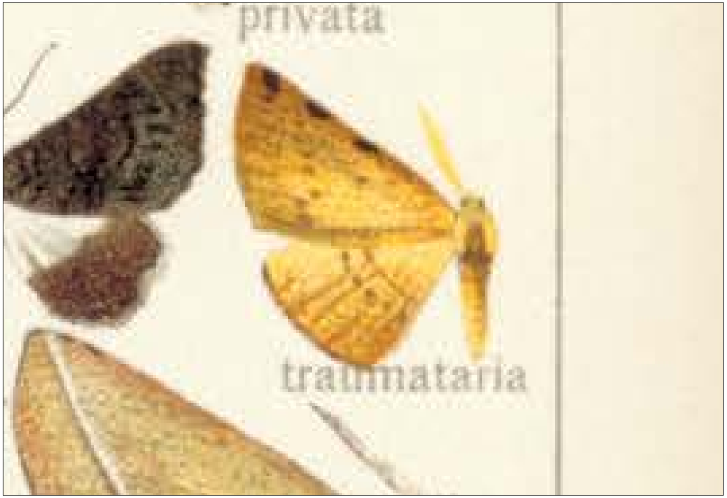

Selected specimen for analysis

EF_Aus-Geo_16 *O. traumataria* Australia (EF)

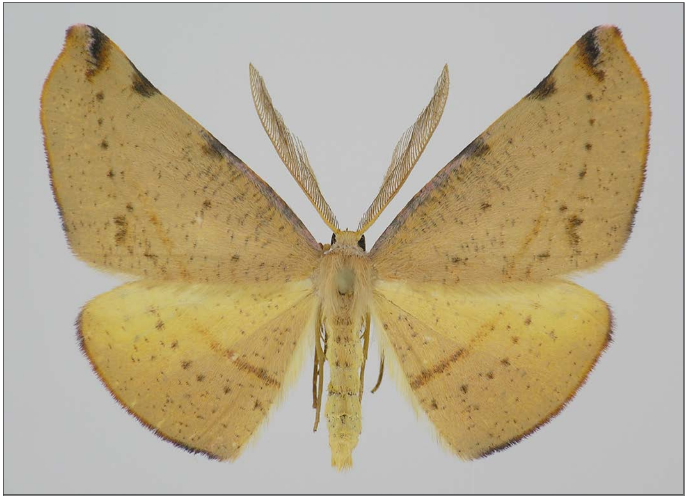

OENO *Parepisparis* Bethune-Baker, 1906 *crenulata* Bethune-Baker, 1906 17 NHM PNG

aus

Comments

7 g

Type photo (if available)

type *crenulata* NHM

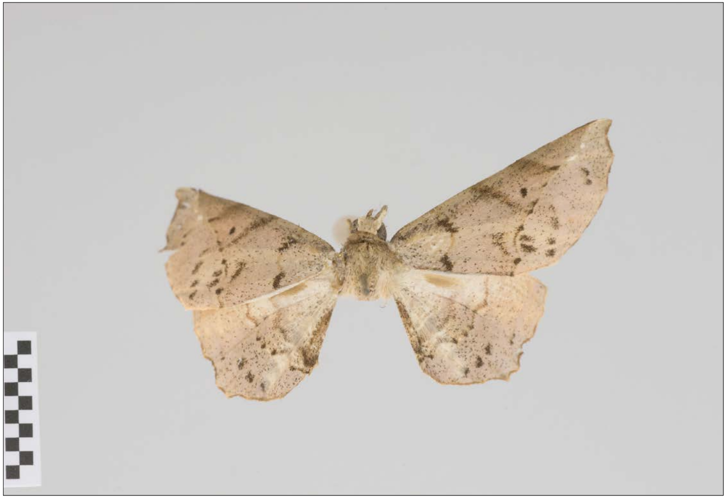

Selected specimen for analysis

EF_Aus-Geo_17 *P. excusata* Australia (EF)

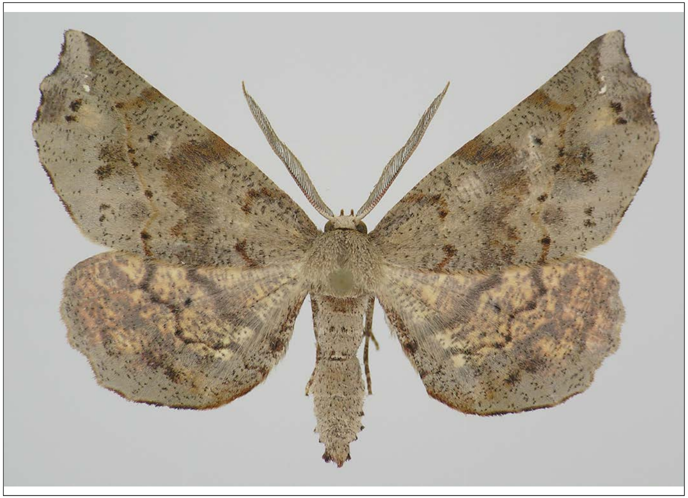

OENO *Sarcinodes* Guenée, [1858] *carnearia* Guenée, [1858] 24 NHM central India aus-oce-ori-pal

Seitz illustration

Comments

Sarcinodes not monophyletic

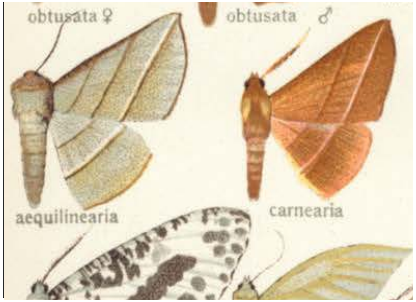

6 g

Type photo (if available)

type *carnearia* NHM

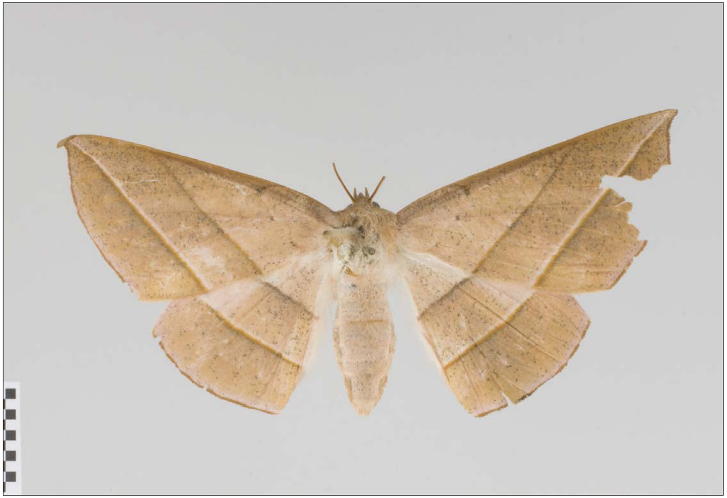

Selected specimen for analysis

PS181 *S. aequilinearia* Thailand (PS)

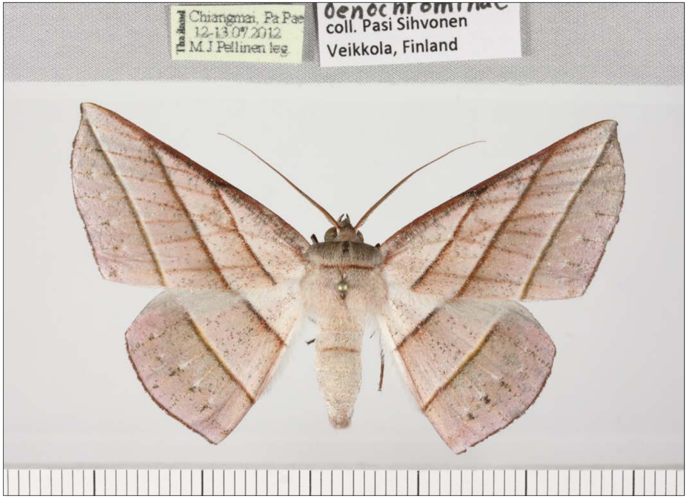

7 New subfamily

Epidesmiinae

OENO > EPID *Abraxaphantes* Warren, 1894 *perampla* Swinhoe, 1890 1 NHM Burma: Upper Tenas- ori

Comments

transferred to Epidesmiinae

5 g

Type photo (if available)

type *perampla* NHM

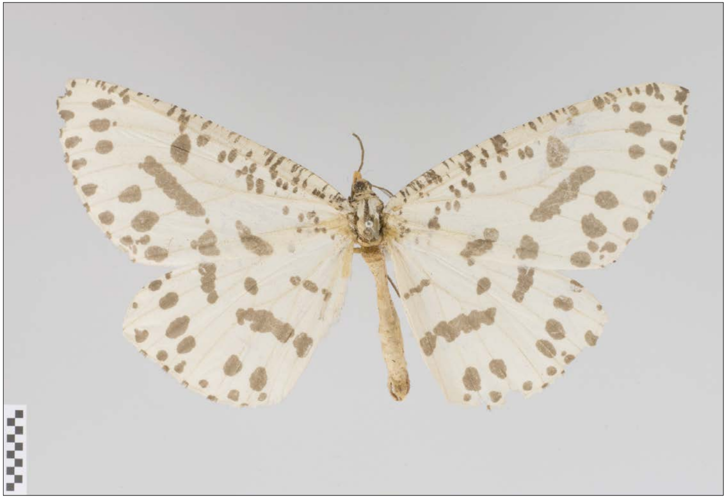

Selected specimen for analysis

PS192 *A. perampla* Thailand (PS)

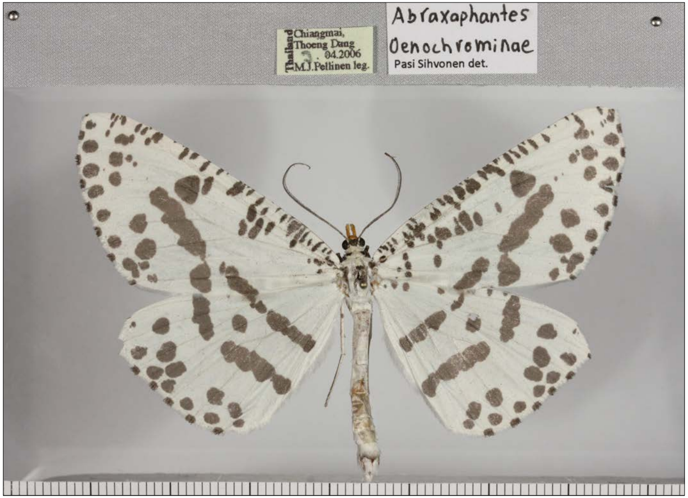

OENO > EPID *Adeixis* Warren, 1897 *insignata* Warren, 1897 5 NHM S Aust-

Comments

transferred to Epidesmiinae

4 g

Type photo (if available)

type *insignata* NHM

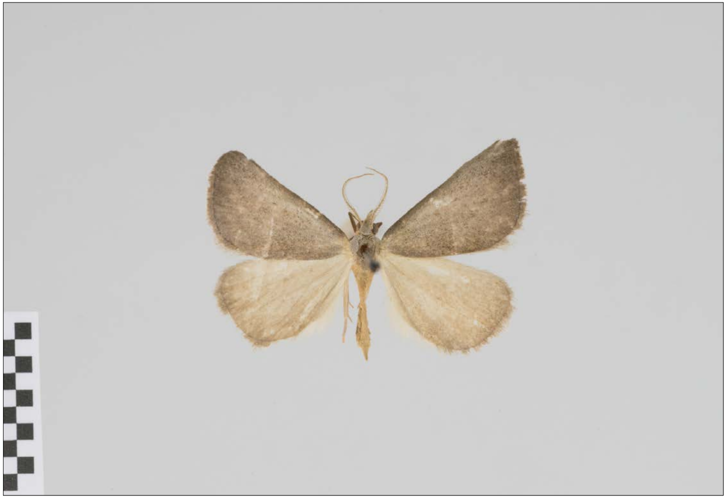

Selected specimen for analysis

MM00246 *A. insignata*

no photograph of analysed specimen

OENO > EPID *Dichromodes* Guenée, [1858] *ainaria* Guenée, [1858] 74 MNHN Tasmania

aus-nz

Comments

transferred to Epidesmiinae

8 g

Type photo (if available)

Seitz illustration *ainaria*

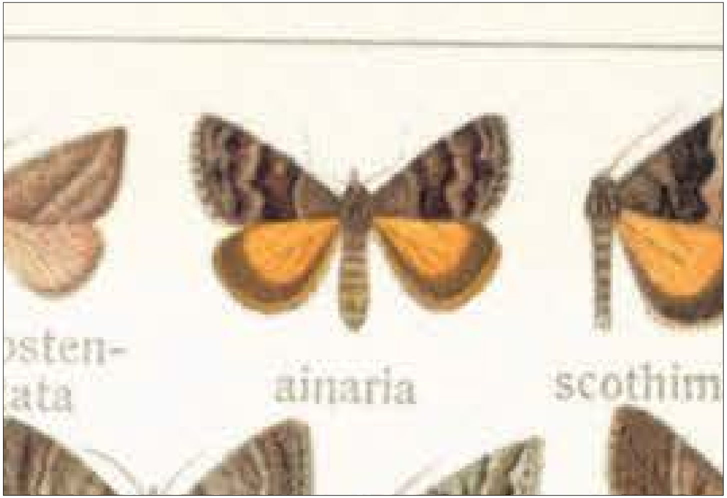

Selected specimen for analysis

EF-Aus-Geo-8 *D. indicataria* Australia (EF)

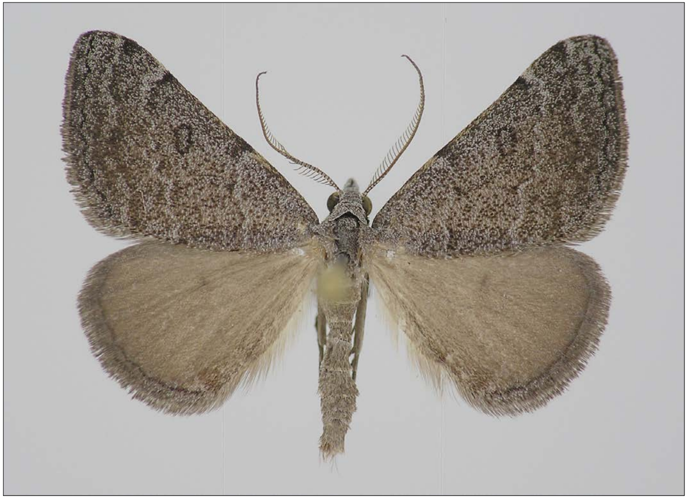

OENO > EPID *Ecphyas* Turner, 1929 *holopsara* Turner, 1929 1 unclear deposit W Australia

aus

Comments

transferred to Epidesmiinae

10 g

Type photo (if available)

no type *holopsara* 10ANIC_11588

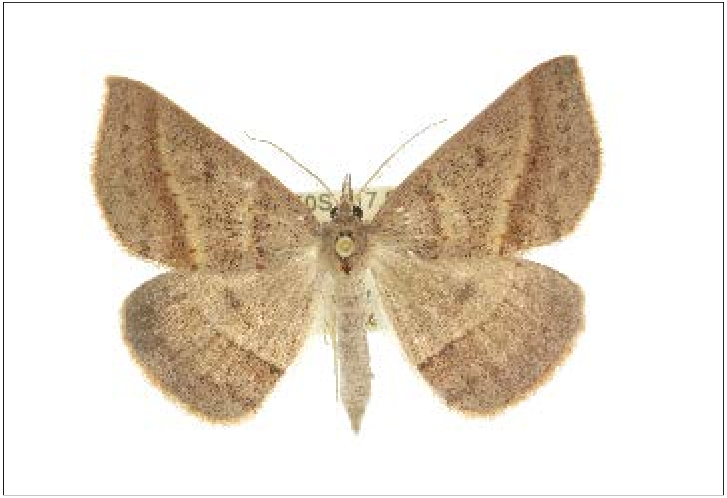

Selected specimen for analysis

EF-Aus-Geo-11 *E. holopsara* Australia (EF)

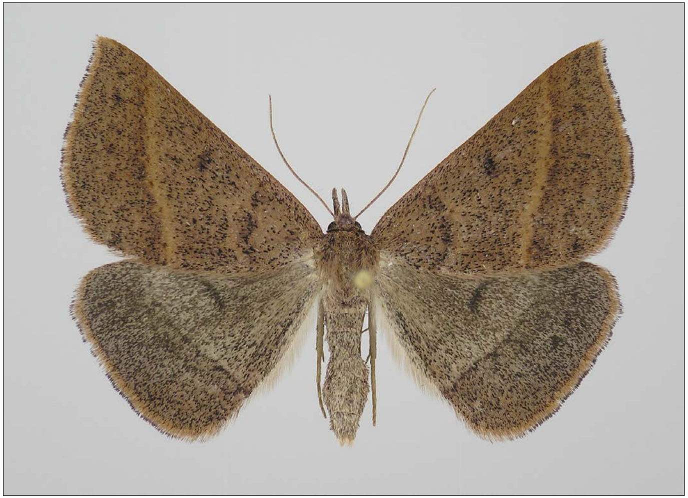

OENO > EPID *Epidesmia* Duncan [& Westwood], 1841 *tricolor* Westwood, 1841 9 OUM Australia: aus

Comments

transferred to Epidesmiinae
type genus and species of new subfamily

8 g

Type photo (if available)

no type *tricolor* NHM

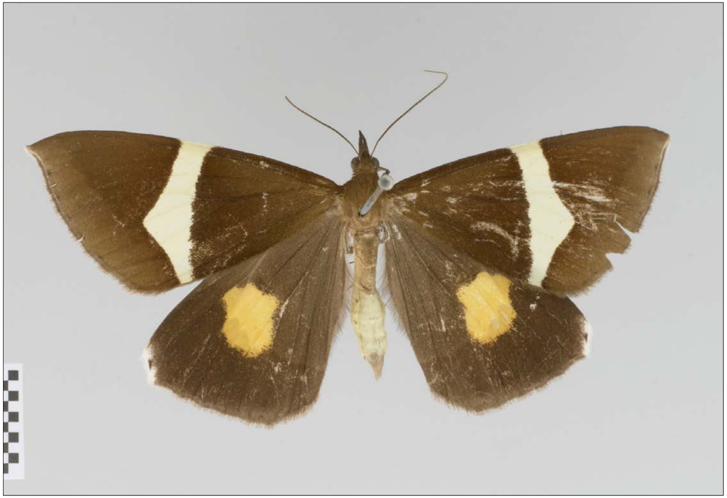

Selected specimen for analysis

EF-Aus-Geo-12 *E. chilonaria* Australia (EF)

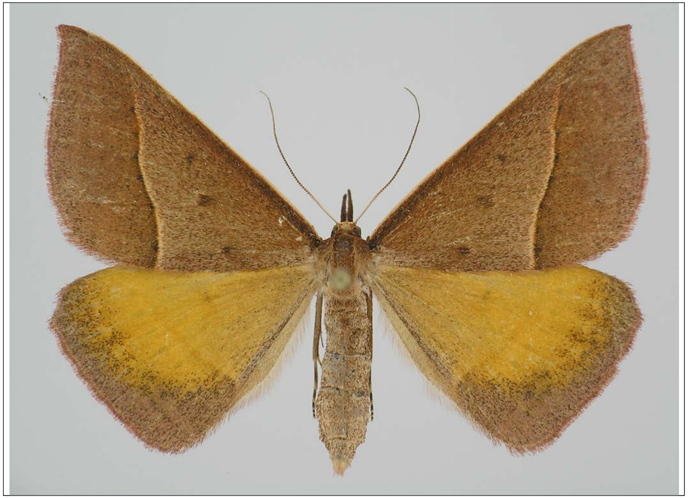

OENO > EPID *Phrataria* Walker, [1863] 1862 *transcissata* Walker, [1863] 1862 4 NHM Australia aus

Comments

transferred to Epidesmiinae

8 g

Type photo (if available)

holotype *transcissata* NHM

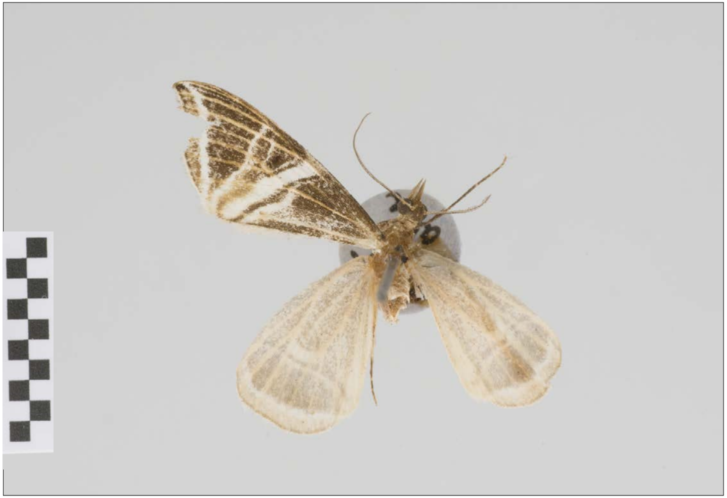

Selected specimen for analysis

EF-Aus-Geo-18 *P. replicataria* Australia (EF)

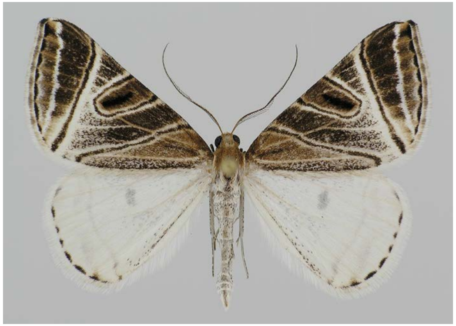

OENO > EPID *Phrixocomes* Turner, 1930 *ptilomacra* Lower, 1892 unclear deposit S Australia aus

Comments

transferred to Epidesmiinae

7 g

Type photo (if available)

no type *ptilomacra* 10ANIC_11643

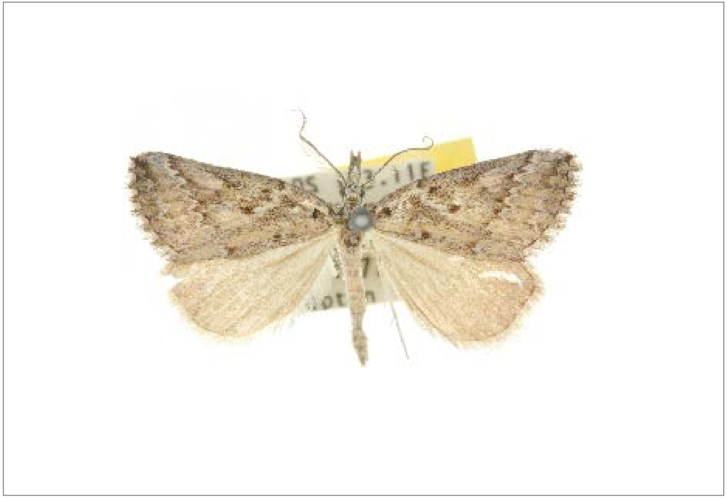

Selected specimen for analysis

EF-Aus-Geo-3 *Phrixocomes* sp Australia (EF)

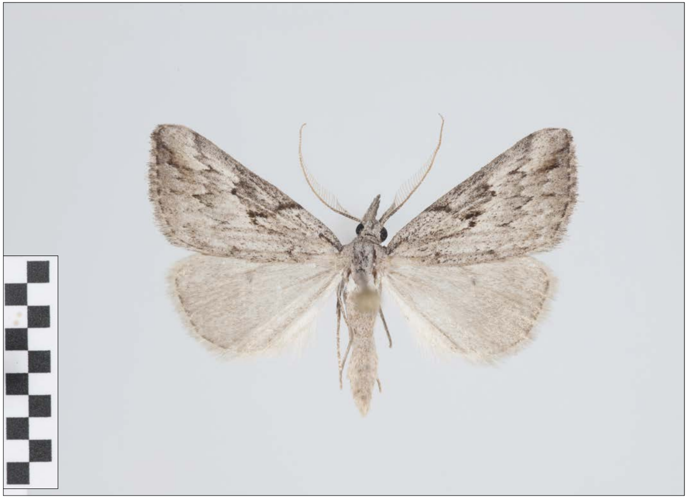

OENO > EPID *Systatica* Turner, 1904 *xanthastis* Lower, 1894 1 unclear deposit Australia QLD aus

Comments

transferred to Epidesmiinae

7 g

Type photo (if available)

no type *xanthastis* 10ANIC_11591

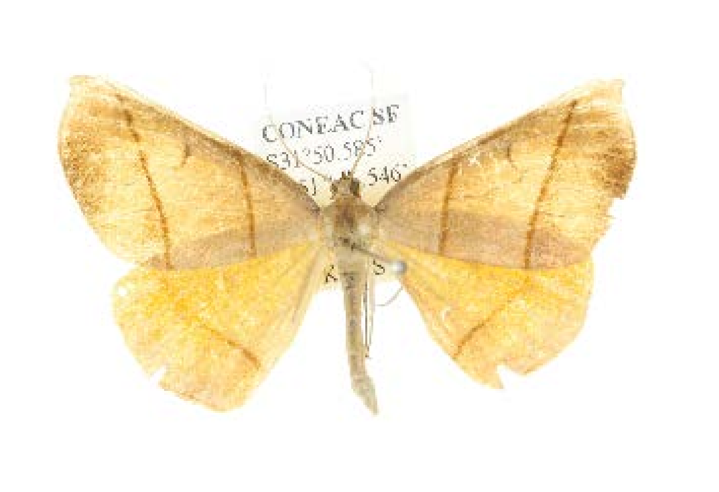

Selected specimen for analysis

EF-Aus-Geo-19 *S. xanthasis* Australia (EF)

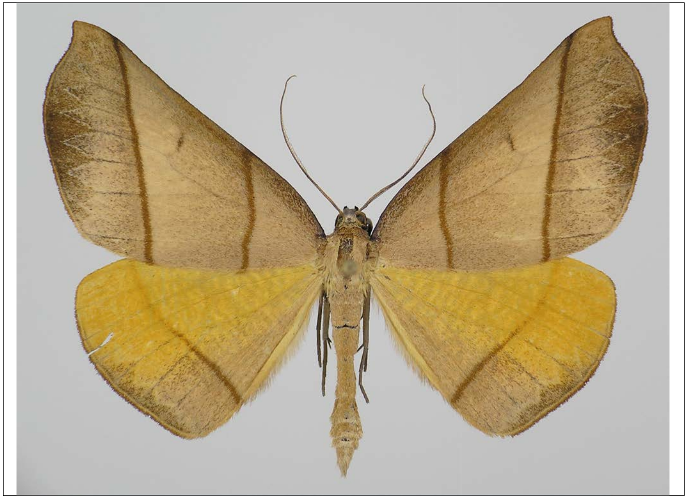

Supplement: Supplemental Information 2 [file peerj-07-7386-s002.pdf]
