# Supplementary material for: A comprehensive molecular phylogeny of Geometridae (Lepidoptera) with a focus on enigmatic small subfamilies
Source: PeerJ. 2019 Aug 27;7:e7386. doi: 10.7717/peerj.7386 (PMC6716565; doi:10.7717/peerj.7386)
Supplement: Supplemental Information 4 [file peerj-07-7386-s004.pdf]

Appendix S4. Adult facies and morphological structures of *Eumelea* Duncan & Westwood, 1841 (Eumeleini).

Fig. s1. **Adult male**. *Eumelea ludovicata* Guenée, 1858. Thailand: Trat, Koh Chang, White [s]and Beach, 24.07.2005, M.J.Pellinen leg. (coll. P. Sihvonen, FIN).

Fig. s2. **8th segment, male**. *Eumelea ludovicata* Guenée, 1858. Thailand: Trat, Koh Chang, White [s]and Beach, 24.07.2005, M.J.Pellinen leg. slide Sihvonen 2746 (coll. P. Sihvonen, FIN).

Fig. s3. **Base of abdomen, male.** *Eumelea ludovicata* Guenée, 1858. Thailand: Trat, Koh Chang, White [s]and Beach, 24.07.2005, M.J.Pellinen leg. slide Sihvonen 2750 (coll. P. Sihvonen, FIN).

Fig. s4. **Uncus, male.** *Eumelea ludovicata* Guenée, 1858. Thailand: Trat, Koh Chang, White [s]and Beach, 24.07.2005, M.J.Pellinen leg. slide Sihvonen 2746 (coll. P. Sihvonen, FIN).

Fig. s5. **Tympanal organ, male.** *Eumelea ludovicata* Guenée, 1858. Thailand: Trat, Koh Chang, White [s]and Beach, 24.07.2005, M.J.Pellinen leg. slide Sihvonen 2750 (coll. P. Sihvonen, FIN).

Fig. s6. **Genitalia, male.** *Eumelea ludovicata* Guenée, 1858 Thailand: Trat, Koh Chang, White [s]and Beach, 24.07.2005, M.J.Pellinen leg. slide Sihvonen 2746 (coll. P. Sihvonen, FIN).

Fig. s7. **Adult female.** *Eumelea* sp. Philippines: Luzon, Camarines sur, Naga Relay Stn., Mount Isarog, Ocampo [no date] (coll. Zoologisches Forschungsmuseum A. Koenig, Bonn, GER).

Fig. s8. **Genitalia, female.** *Eumelea* sp. Philippines: Luzon, Camarines sur, Naga Relay Stn., Mount Isarog, Ocampo [no date]; slide Sihvonen 2761 (coll. Zoologisches Forschungsmuseum A. Koenig, Bonn, GER).

Fig. s9. **Wing venation, male.** *Eumelea ludovicata* Guenée, 1858. Thailand: Trat, Koh Chang, White [s]and Beach, 24.07.2005, M.J.Pellinen leg. Forewing slide Sihvonen 2748, hindwing slide Sihvonen 2749 (coll. P. Sihvonen, FIN).

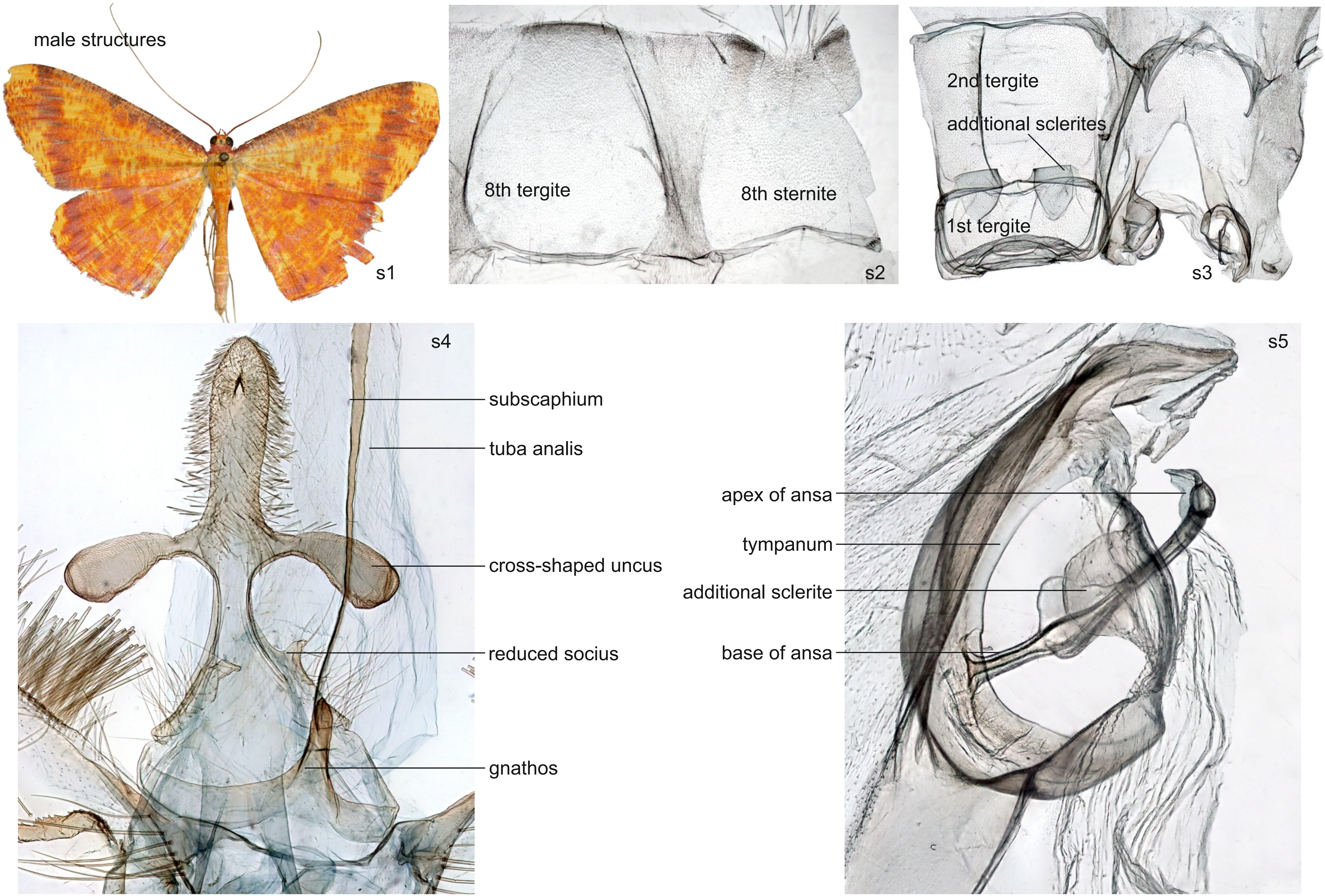
male structures
s1
8th tergite
8th sternite
s2
2nd tergite
additional sclerites
1st tergite
s3
s4
subscaphium
tuba analis
cross-shaped uncus
reduced socius
gnathos
s5
apex of ansa
tympanum
additional sclerite
base of ansa

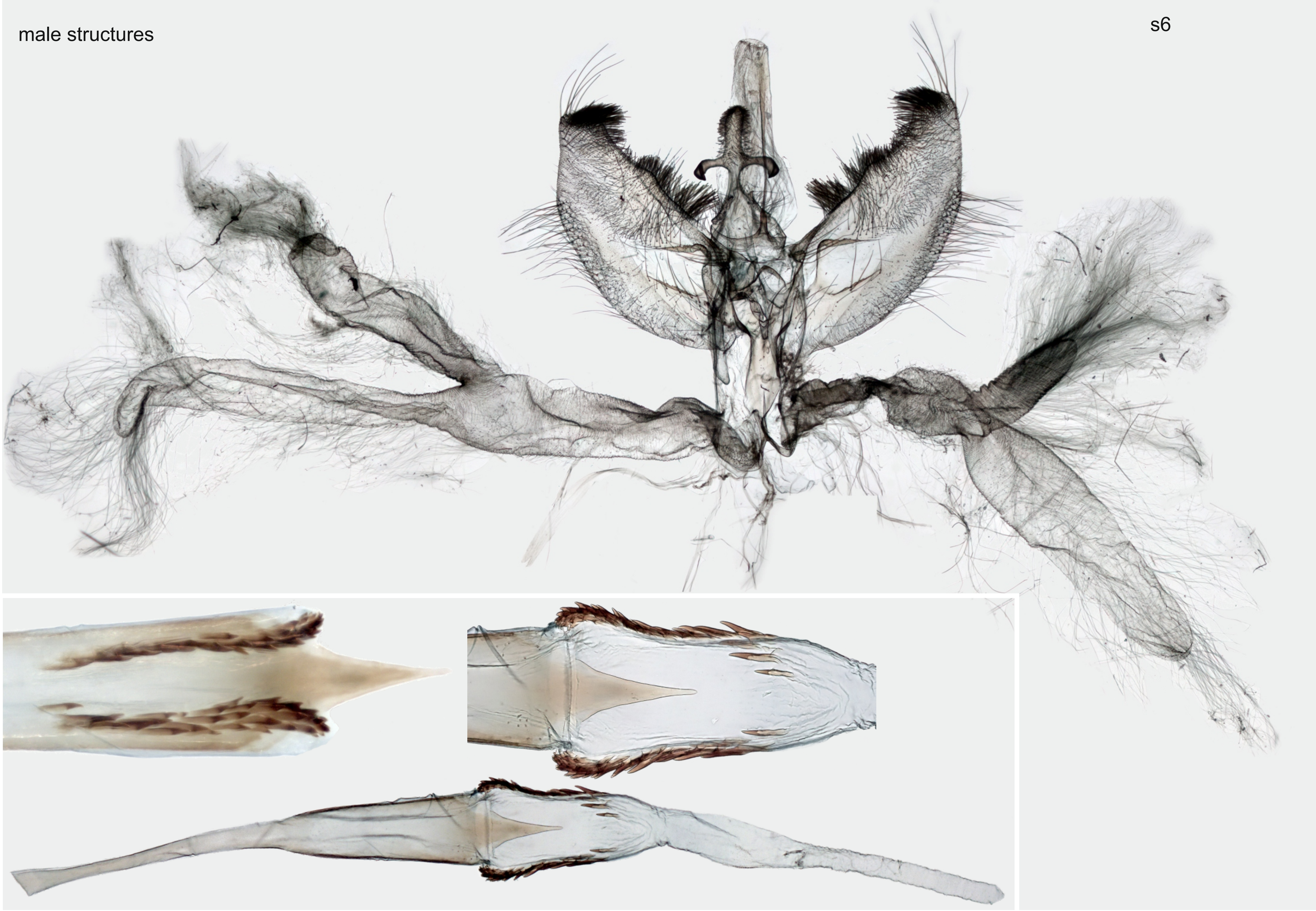
male structures
s6

female structures

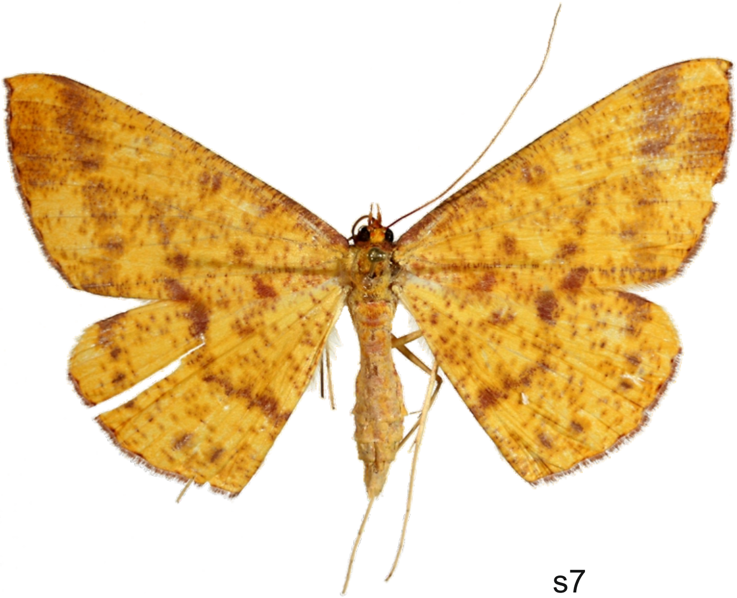

s7

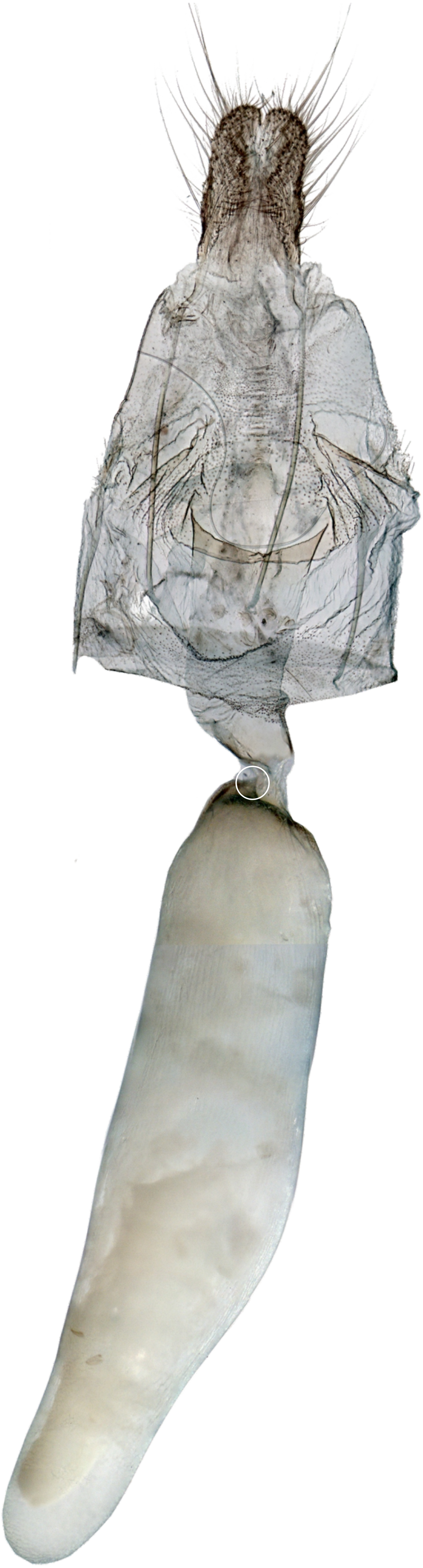

s8

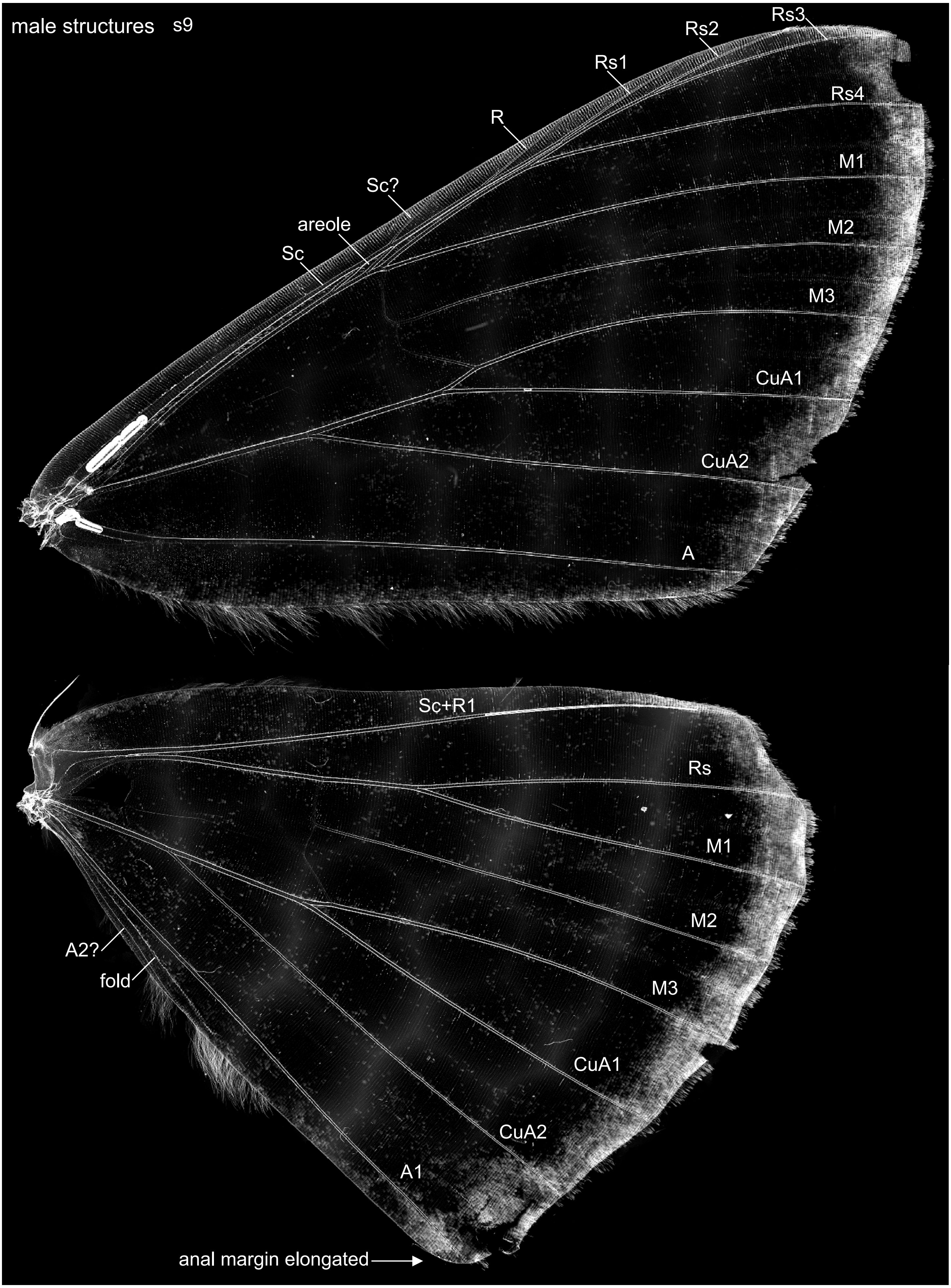
male structures s9
Rs3
Rs2
Rs1
Rs4
R
M1
Sc?
areole
M2
Sc
M3
CuA1
CuA2
A
Sc+R1
Rs
M1
M2
A2?
fold
M3
CuA1
CuA2
A1
anal margin elongated
