# Supplementary material for: A comprehensive molecular phylogeny of Geometridae (Lepidoptera) with a focus on enigmatic small subfamilies
Source: PeerJ. 2019 Aug 27;7:e7386. doi: 10.7717/peerj.7386 (PMC6716565; doi:10.7717/peerj.7386)

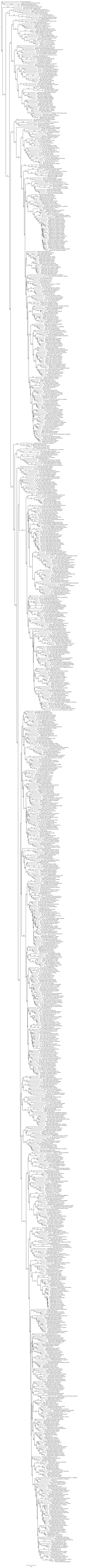

Supplement: Supplemental Information 3 [file peerj-07-7386-s003.pdf]
